# Graph-Based Resource Allocation for Integrated Space and Terrestrial Communications

**DOI:** 10.3390/s22155778

**Published:** 2022-08-02

**Authors:** Antoni Ivanov, Krasimir Tonchev, Vladimir Poulkov, Agata Manolova, Nikolay N. Neshov

**Affiliations:** Faculty of Telecommunications, Technical University of Sofia, bul. Kl. Ohridski 8, 1000 Sofia, Bulgaria; k_tonchev@tu-sofia.bg (K.T.); vkp@tu-sofia.bg (V.P.); amanolova@tu-sofia.bg (A.M.); nneshov@tu-sofia.bg (N.N.N.)

**Keywords:** 6G, device-to-device networks, cellular networks, cognitive radio, graph, heterogeneous networks, resource allocation, wireless communications

## Abstract

Resource allocation (RA) has always had a prominent place in wireless communications research due to its significance for network throughput maximization, and its inherent complexity. Concurrently, graph-based solutions for RA have also grown in importance, providing opportunities for higher throughput and efficiency due to their representational capabilities, as well as challenges for realizing scalable algorithms. This article presents a comprehensive review and analysis of graph-based RA methods in three major wireless network types: cellular homogeneous and heterogeneous, device-to-device, and cognitive radio networks. The main design characteristics, as well as directions for future research, are provided for each of these categories. On the basis of this review, the concept of Graph-based Resource allocation for Integrated Space and Terrestrial communications (GRIST) is proposed. It describes the inter-connectivity and coexistence of various terrestrial and non-terrestrial networks via a hypergraph and its attributes. In addition, the implementation challenges of GRIST are explained in detail. Finally, to complement GRIST, a scheme for determining the appropriate balance between different design considerations is introduced. It is described via a simplified complete graph-based design process for resource management algorithms.

## 1. Introduction

With the fifth generation (5G) of wireless communications becoming more prevalent for service provision in a plethora of civil, medical, and industrial applications (such as Internet of Things (IoT), Augmented Reality, Virtual Reality, Holographic communications, Industry 4.0, and eHealthcare [1,2,3]), the spectrum utilization efficiency will be of greater importance than the availability of additional frequency bands as networks become increasingly dense and heterogeneous. It has been projected that the global mobile traffic per month will increase over one-hundred-fold (having already grown to over 50 exabytes) by the end of this decade, with the monthly traffic per user growing with an even faster rate, reaching about 250 gigabytes [2]. Moreover, the global number of user terminals is expected to surpass 100 billion. In order to accommodate new bandwidth-intensive applications and the very significant upsurge in the number of user equipment (UEs) devices beyond 5G and 6G, inter-connectivity and resource sharing between the communication networks on the ground, at sea, in the air, and in space will be required [2]. Addressing such tremendous challenges will require the implementation of mechanisms for efficient RA (considered to be implemented through methods based on graph theory within the scope of this review) [3]. From a design point of view, the many communication technologies and applications conceptualized for 6G, depend, in general, on the implementation of this functionality. It provides the essential mechanisms for wireless communication in future standards, even though their definitions and solutions vary in accordance with the deployment scenario, the devices’ capabilities, and quality-of-service specifications [2,3]. The RA procedure is responsible for adequate distribution of the network’s very limited resources (frequency channels and/or transmission power) in a complex radio propagation environment, and with the increasing number of UEs, in view of reducing the interference, which is usually unavoidable. Furthermore, the densification of the network’s nodes creates potential for considerable overhead that would result in intolerable delays [4]. Consequently, the RA scheme has to actively minimize it during the network’s operation, while providing optimal spectral efficiency (maximizing the throughput gains on the available spectrum) at the same time. Due to the various nodes deployment densities in different networks, the RA procedures differ in requirements and complexity. It is notable that the RA functionality is usually applied for one (or a combination) of the following three wireless network scenarios (they are also considered in recent, more general surveys in the field [4,5,6,7,8], which review machine learning and heuristic methods for RA) within the context of 5G and beyond:

- Homogeneous and heterogeneous networks (Het-Nets). Most current wireless communication systems are of this type. Traditionally, terrestrial Het-Nets consist of cells with various ranges that serve multiple mobile UEs within their area of coverage. With the advent of integrated space and terrestrial networks (ISTNs) [2], Het-Nets expand to incorporate multiple ground-based licensed/license-free cellular networks, cell-less ultra-dense networks, as well as communications between unmanned aerial vehicle (UAV) based, marine, and satellite nodes. When only one communication standard is used in the network’s nodes, it is homogeneous. The base stations (BSs) are usually stationary, but they can also be mobile. Users are often characterized by bandwidth-intensive applications, and attach to the cell (or cells) which provides the most favorable throughput. RA is predominantly focused on providing inter-cell interference (ICI) coordination.
- Device-to-Device (D2D) Networks. They are characterized by the fact that the UEs themselves provide radio access for each other, and their communication exchanges are not intermediated by the BSs/Access Points (APs). Accordingly, low delays and high throughputs can be achieved, which makes D2D communications more prevalent in modern networks. The most significant challenge for them is what spectrum they are to utilize, considering D2D communications usually do not have particular bands assigned to them; instead, they complement the networks which use either cellular or unlicensed spectra [6].
- Cognitive Radio (CR) Networks. They increase the utilization of the spectrum allocated to other previously deployed wireless systems by accessing it opportunistically (i.e., only at locations, periods of time, and frequency channels in which no transmissions from the incumbent users, are present). CR-enabled UEs and APs (cognitive APs or CAPs) can determine the spectrum availability and perform DSA independently from, or in conjuncture with, the primary network.

Each of these types of networks can be used in conjuncture with each other, and as technologies which are used as a basis for the implementation of a specific network, or its integration with already deployed networks [6]. Then, as they increase in the number of connected devices and complexity, it becomes more difficult to achieve their primary communication objectives, namely (1) interference reduction; (2) spectrum utilization; and (3) spectral efficiency.

For a long time, graph theory has been used to define, as well as to relax, optimization problems in telecommunications. It is also an appropriate instrument for solving these tasks, because wireless nodes and their parameters (type, frequency resources, location, transmission power and interference limits, and computational capabilities) can amply be represented as a graph. Some general communication problems that have utilized graph methods include (but are not limited to) RA and transmission power control in cellular networks, beamforming, link scheduling, traffic prediction, channel estimation, localization, cooperation and information transfer between vehicles for autonomous driving, compression of point clouds for transfer of images, UAV trajectory control for throughput maximization, detection of unauthorized traffic and of its sources, user association (UA), cooperative caching of data between D2D wireless nodes, wired networks configuration and communication delay analysis, and encrypted traffic classification [9,10,11,12].

### 1.1. Literature Search and Selection Methodology

**Figure 1:**
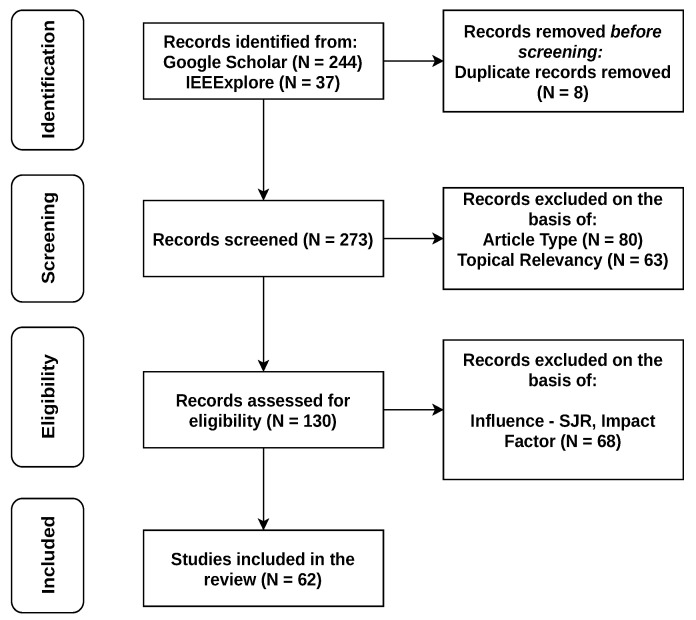
Summary of PRISMA flowchart of the article selection process for this review.

This paper specifically surveys graph-based RA algorithms categorized in each of these network types in a separate Section. This classification is based on the significant number of relevant papers published in highly influential journals and conferences in recent years (60% of those since 2017). The overall number of papers (out of 130 relevant works, published after the year 2006) chosen to be included in this review, after filtering of their contents and topics in regards to relevance and influence, is 62, which shows that the topic of graph-based RA methods is still very open to further investigation. The process of relevant literature selection for this survey follows the PRISMA protocol [13], and is illustrated in Figure 1. This is further highlighted by the analysis of recent relevant surveys in Section 2.

### 1.2. Article Structure

**Figure 2:**
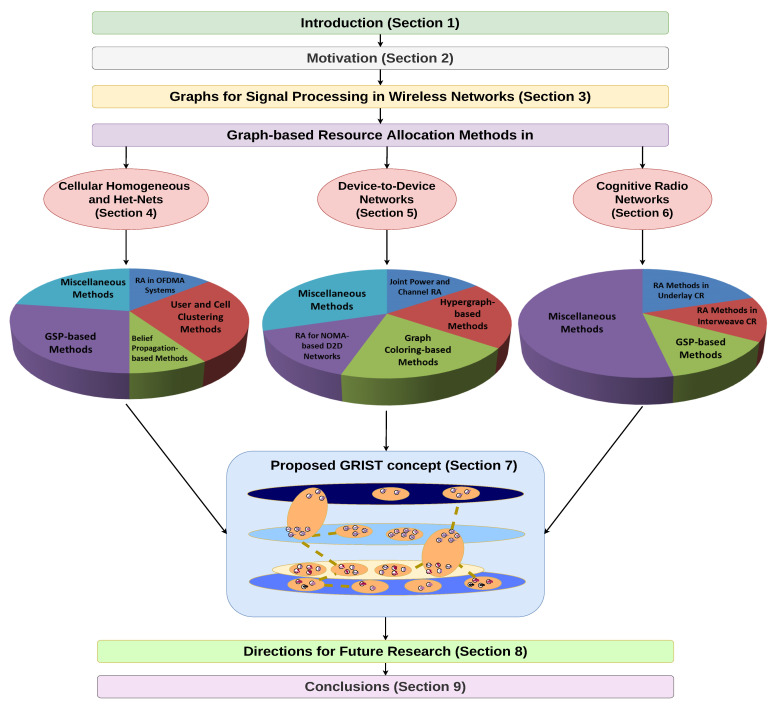
Graph-based RA in advanced wireless networks.

The structure of this article is illustrated in Figure 2. Section 1 contains the introduction to the topic. A motivation for why its contribution is significant is provided in Section 2. Then, Section 3 reviews the essential characteristics and types of graphs for the application of such methods in wireless communications. The reviewed graph-based RA methods are classified in the three categories of network types (homogeneous and Het-Nets, D2D and CR), each in a separate Section. For each category, there are sub-categories (each representing a Subsection within the Sections), defined as common features of research that include papers with similar problem formulations or system model scenarios. These sub-categories are presented in Figure 2 as pie-charts to illustrate their relative influence within each category. The volume of each sub-category in the chart is related to the number of papers that are reviewed in it. In each Section, particular emphasis is placed on the way in which the optimization problems are defined via graphs, and in what manner they are solved. This is an important consideration for such problems, because they need to be formulated through appropriate graph structures that usually are not trivial to define. On the basis of the review of the graph-based RA methods for the three categories of networks in Section 4, Section 5 and Section 6, the GRIST concept is introduced in Section 7. Its implementation challenges and a graph-based model for algorithm design are also introduced.

The contributions of this article can be summarized as follows:

- The methods for RA are categorized according to their network scenario (Het- Net/D2D/CR). The graph-based solutions and their performance are thoroughly surveyed. For each category, a tabular outline of the main design characteristics (such as the graph type and its formulation, and the optimization method used for the solution) is given. Analysis of these characteristics provides features of the research in every type of network scenario, as well as directions for future development.
- On the basis of the surveyed methods and existing visions for 6G ISTNs, the concept for Graph-based Resource allocation for Integrated Space and Terrestrial communications (GRIST) is introduced. It conceptualizes the design paradigm for the interconnectivity and coexistence of different networks within ISTN by describing their properties via a hypergraph and its attributes. The challenges in implementing GRIST are also given. Then, the design process of RA algorithms in GRIST is itself modeled by a simplified variant of a complete graph, thus presenting a scheme for determining the appropriate balance between the different design aspects (such as delay and computational complexity).

Lists of acronyms and notations, with their definitions, are given in Table 1, Table 2, Table 3 and Table 4, respectively. The rest of this article is organized as follows. The findings of relevant survey papers are summarized in Section 2, to illustrate the motivation for this work. The main types and properties of graphs, as well as their capabilities for signal processing in telecommunications, are provided in Section 3. Graph-based methods for RA in homogeneous and Het-Nets, D2D, and CR networks are reviewed in Section 4, Section 5 and Section 6, respectively. The proposed GRIST concept, challenges for its implementation in 6G ISTNs, as well as the model for the design process of RA algorithms are conceptualized in Section 7. Several directions for future research in the area are identified in Section 8. Lastly, the conclusions of this article are summarized in Section 9.

## 2. Motivation

In the recent years, substantial research efforts have been made to develop different kinds of algorithms for RA, including those that utilize graph-based design, for various network scenarios. To the best of the authors’ knowledge, there are no other surveys in the literature that aim attention directly at graph-based RA, but there are others that are relevant to the present analysis. This section reviews contemporary surveys in the field and briefly explores the topics that they are focused on, as well as their limitations, which are summarized in Table 5. Accordingly, the contributions presented in this article are additionally emphasized. A comprehensive survey of deep-reinforcement-learning (DRL)-based solutions for RA in Het-Nets is presented in [5]. The advantages of employing DRL (the chief of them being that they better facilitate the solution of the complex optimization problems involved) in contrast with non-learning algorithms, as well as challenges for future research, are described. Categorization is performed of both the types of Het-Nets, and of the relevant techniques for DRL employed in the literature. Nonetheless, graph-based solutions are not considered. A broad survey of graph neural network (GNN)-based algorithm designs for various procedures in wireless communications and image processing is presented in [11]. The basics of the most commonly used GNN structures and their applications in telecommunications are described. Key issues in employing these algorithms are outlined; however, the discussion on RA is limited only to those that are based on GNNs. The article [12] presents a more expansive view on the applications of GNNs by including problems in not just wireless, but also wired and software-defined networks. For each kind of network, the relevant problems and the types of GNNs are categorized. Nevertheless, the discussion on RA based on GNNs is limited. The authors in [4] provide a comprehensive survey of the challenges related to RA in the different types of Het-Nets. Consequently, the analytical definitions of wireless system capacity for each type are presented, and methods for solving the RA problem are reviewed. An in-depth classification of the RA algorithms is made, according to the tasks they complete (such as UA and beamforming), and the performance parameters by which they are evaluated. Finally, the authors propose two paradigms for RA in future cellular Het-Nets. Advancements in D2D communications are thoroughly analyzed in [6]. The modes of implementation and integration of D2D nodes with existing and upcoming networks within the context of 5G are explored in detail, together with several important factors for their realizations (such as device discovery, RA, security, economics-related benefits, and others). These aspects represent the categories in which the literature is summarized, and for each of them, general solutions and relevant challenges are outlined. Furthermore, a general review of machine learning (ML)-based algorithms for CR networks is made in [8]. It is focused on various ML methods for spectrum sensing, dynamic spectrum allocation, and spectrum prediction, and the relevant research challenges are outlined.

*In summary, these surveys offer limited discussion of the design and implementation of graph-based RA methods in contemporary wireless networks, thus providing ample motivation for the analysis made in this article*.

**Table 5:** Comparison of relevant surveys on graph methods applied in wireless communication networks.

Survey Paper	Topics Addressed	Principal Advantages	Limitations, Relevant to This Work
Alwarafy et al. (2021) [5]	DRL algorithms for RA and UA in Het-Nets	Categorization of Het-Net scenarios and of the DRL methods used; advantages of DRL	Graph-based algorithms are not discussed
He et al. (2021) [11]	GNN-based solutions for wireless communications and image processing	Key challenges are outlined	Limited discussion of RA
Jiang (2021) [12]	GNN-based solutions for telecommunication networks	Comprehensive survey in wired, wireless, and software-defined networks	Limited discussion of RA
Xu et al. (2021) [4]	RA methods in Het-Nets	Categorization of RA algorithms according to their relevant tasks and performance parameters; new models for RA are proposed	Limited discussion of graph-based RA methods
Jameel et al. (2018) [6]	Aspects of D2D communications	Categorization of methods for implementing the main implementation aspects of D2D	Limited discussion of graph-based RA
Upadhye et al. (2021) [8]	ML-based algorithms for spectrum sensing and RA in CR networks	Categorization of ML methods according to the design for spectrum sensing, prediction, and allocation	Limited discussion of graph-based RA methods
**This paper**	Graph-based RA algorithms for RA in homogeneous and Het-Nets, D2D, CR and ISTNs	Comprehensive survey of the design and performance of graph-based RA algorithms; discussion of challenges for graph-based design for inter-connectivity and coexistence algorithms in ISTNs through GRIST	-

## 3. Graphs for Signal Processing in Wireless Networks

Applying Graph Theory to signal processing has recently attracted significantly more attention in the Telecommunications Engineering community, which is emphasized by the successful implementation of such approaches for diverse problems in the areas of services supplied via social networks, cognitive radio, and vehicular networks organization, and more [14,15]. These and other potential applications require rigorous analysis of the different domains which are involved in the explored data’s generation. The graph consists of three primary elements: vertices, edges, and directivity (the last one being optional). Vertices are the data points on a graph, and they usually carry information about the amplitude/value and physical position of the signal measurement sample. The vertices are labeled according to their physical meaning, and therefore the labels are not necessarily ordered numbers. The edges connect these vertices, which are related to each other in some domain. Their relationships between two vertices can be binary (0—they are not related and thus, there is no edge between them, and 1—otherwise) or variable (each existing relationship has a certain weight (weighted graphs)). Should there be any directionality in the way vertices are organized (hence introducing yet another information domain), arrows are placed on the edges to signify the direction in which two data points are related to one another. Such graphs are directed, whereas their alternatives are called undirected.

A graph is analytically defined as G={V,B}, i.e., a set of vertices V connected by the edges contained in the set B, such that B⊂V×V, × being the direct product operator. The relationship (edge) between vertices (nodes) *m* and *n* is described as (m,n)⊂B. The complete set of B is composed of all connections between the vertices which are related to each other. In an indirected graph, the connections are in both directions, whereas for the directed, they are made in a specified direction. In order to describe the way in which the sets of vertices and edges are related, a number of matrices are used to find solutions inspired by the relationships in different domains modeled via graphs [16]:

- Adjacency matrix A. The most important mathematical description of the graph, the adjacency matrix of size N×N (*N* is the number of vertices) which describes the connectivity between edges and vertices. The columns of A show the connections of each vertex to the rest.
- Incidence matrix B. It is similar to the adjacency matrix, but its dimensions are M×N (*M* is the number of edges). For undirected graphs, there are ones on all positions which indicate a vertex connected to an edge.
- Weight W matrix. Its dimensions and the positions of the non-zero elements match those of the adjacency matrix. The weight matrix can be obtained by replacing the non-zero values of A with coefficients (the edges’ weights) that carry physical meaning in some domain and are defined according to it.
- Degree matrix D. Its dimensions are N×N, and it contains the sum of the weights of all edges connected to each of the vertices. So D shows that the importance of each vertex in the overall graph structure can be determined.
- Laplacian matrix L. This is derived from the matrices above and is significant for practical signal processing applications. The Laplacian matrix is defined as L=D−W. Its dimensions are N×N, and it is characterized by positive elements on the diagonal and negative on the off-diagonal non-zero elements.

Some metrics that arise from the connected structure of the graph are the following. The series of linked nodes and edges between any two vertices (as an example, *i* and *l*) is called a *walk*. The sum of the edges between these two vertices along a particular walk is its *length*. The adjacency matrix $A^{K}$ of size N×N contains the number of walks of length *K* between any two vertices. A *K*-*neighborhood* of a vertex is comprised of the vertices which are up to *K* walks away from that vertex. A walk which includes any vertex along its route only once, is a *path* within the graph. The number of edges included in the path is its length (cardinality) and the sum of their weights is the path weight.

### 3.1. Types of Graphs

Several types of standard graph models [16] relevant for the analysis in this article are described below.

**Figure 3:**
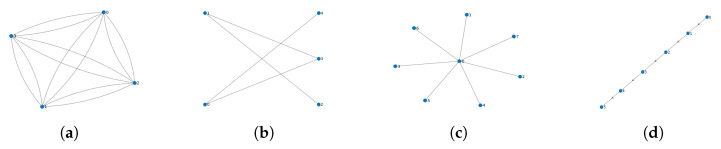
General graph structures relevant to RA methods. (**a**) Complete graph. (**b**) Bipartite graph. (**c**) Star graph. (**d**) Path graph.

- General/Complete graph—This category includes various kinds of graphs that are defined using the physical relationships between the wireless network’s nodes (such as interference, social connections between users, and others). As for the complete graph, each of its vertices is connected to all of the rest. Accordingly, all non-diagonal entries of A will be one, and all of the diagonal ones will be zero. An example is illustrated in Figure 3a. Depending on the physical relationships described via the model, some of the graphs are not necessarily complete.
- Bipartite (Kuratowski) graph—its vertices can be divided into two subsets H and E, such that there are edges between the nodes of each subset, but not those within the subset (Figure 3b). If all vertices in H are connected to those in E, the bipartite graph is complete. This type of graph may be extended to more than two subsets (a tripartite graph, for example, is composed of three subsets—two on each side and one between them), the vertices of each being connected to those of the adjacent subset.
- Star graph—a bipartite graph for which one subset contains only one vertex, while the other consists of the remaining vertices. In this way, the graph is formed in the shape of a star (Figure 3c) with one vertex connected to all of the others, whereas they do not have connections between themselves.
- Path graph—its first and last vertices are connected to only one other vertex (i.e., they have a degree of connectivity J=1), whereas all the rest connect to two (J=2). A path graph is directed if all of its edges have directions which point to the succeeding vertices (Figure 3d).

### 3.2. Signal Processing on Graphs and Graph Neural Networks

The input datum (generally referred to as a signal) is processed through filtering by one of the matrices which describe the graph’s structure (usually the Laplacian or the degree matrix). Another common term for filtering is graph shift, because the graph’s matrix shifts the signal’s samples around the vertices of the graph [16,17]. This operation (similar to data analysis in the time and frequency domains) can be described in either the vertex (analytically analogical to the time domain analysis), or the spectral (the Fourier transform of the vertex domain signal) domains. The fundamental expression of graph signal processing (GSP), given in the vertex domain, is:

(1) $$y=h_{0}A^{0}x+h_{1}A^{1}x+\cdots +h_{M-1}A^{M-1}x=\sum_{m=0}^{M-1}h_{m}A^{m}x\;,$$

where a signal x is filtered (or shifted) by a graph with an adjacency matrix A by m=0,…,M−1 filters, i.e., *M* different shifts are applied to the signal (corresponding to *M* stages of a filter), and their products are combined to obtain the resultant signal y, while $h_{0},\ldots ,h_{M-1}$ are the scaling coefficients. The graph discrete Fourier transform (GDFT) of the signal x is expressed as $X=U^{-1}x$, where X is the vector of GDFT coefficients, while $U^{-1}$ is a matrix composed of the column eigenvectors of A. Correspondingly, the eigen-decomposition of the adjacency matrix is as follows: $A=U\Sigma U^{-1}$, Σ being the matrix of eigenvalues of L that is used to express the graph filtering equation in the spectral domain:

(2) $$Y=\sum_{m=0}^{M-1}h_{m}\Sigma^{m}X\;.$$

Solving the problem of finding the filter’s coefficients that minimize the $l_{2}$-norm error is commonly achieved via the Taubin α−β algorithm. The parameters α and β, which determine the optimal solutions, are found empirically. This method is well illustrated through examples in [14,17].

GNNs combine GSP and the feature-extraction properties of traditional neural networks to allow for the efficient analysis of non-Euclidean data, as is represented via graph structures. These model-learning approaches are based on extracting information from the attributes/features of a graph’s vertex, as well as the vertices with which it is connected through edges (which may also have separate attributes) [11,18]. By aggregating them via a particular mathematical operation (most often convolution), a hidden representation of this vertex is produced. Each GNN layer processes the input data (attributes matrix) $X\in R^{M\times S}$, i.e., *M* vertices with *S* attributes each, in a manner similar to (1), through the matrix $U_{L}$ composed of the column eigenvectors of the Laplacian matrix L and the neuron weights matrix W. As an example, the output of the *k*-th layer $X_{:,j}^{k}$ of a convolutional GNN is defined thus:

(3) $$X_{:,j}^{k}=F\left[\sum_{i=1}^{C-1}U_{L}\;W_{i,j}\;U_{L}^{T}\;X_{:,i}^{k-1}\right]\;,\;j=1,\ldots C\;,$$

where *C* is the number of the layer’s output channels (consequently, C−1 is the number of input channels) and F is the layer activation function. Other notable types of GNN are graph attention networks, graph auto-encoders, graph recurrent networks, and spatial–temporal GNNs [11].

## 4. Graph-Based RA in Cellular Homogeneous and Het-Nets

**Figure 4:**
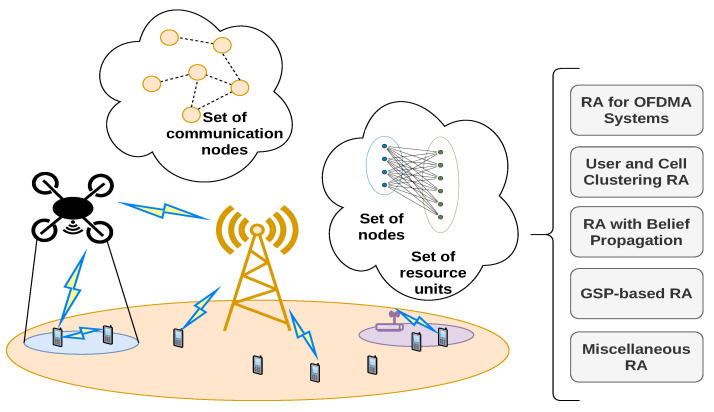
Scenario of homogeneous and Het-Nets and summary of methods for graph-based RA.

Most algorithms which utilize graphs are applied for networks of this type, due to their continual prevalence in modern wireless communications. Even with the roll-out of 5G, most mobile operators still rely heavily on macro cells, or their combination with small cells of different range. They need to achieve efficient resource distribution and interference reduction, as the number of users and per-user throughput increase. This section analyzes the development of graph-based RA algorithms in throughput-intensive cellular networks, mostly in the context of the fourth generation of mobile communications. The scenario of homogeneous and Het-Nets is illustrated in Figure 4. It also includes a generalized illustration of the most common graph models for RA problems (usually used independently from each other), and a summary of their subcategories to comprise this Section. A tabular summary of the main design characteristics (such as the graph type and its formulation, and the optimization method used for the solution) of the algorithms reviewed in this section, is given in Table 6.

### 4.1. RA Methods in OFDMA Systems

Most modern cellular networks utilize the orthogonal frequency division multiple access (OFDMA) scheme, and therefore, substantial research efforts have focused on optimizing its efficiency for RA. The authors of [19] propose a solution which assigns users spatial channels for all OFDMA symbols in a downlink (DL) frame according to each user’s particular bit error rate and throughput requirements. A single-cell network serving a variable number of users, with a fixed number of channels, is considered. The channel state is assumed as constant (stationary/slow moving UEs) and ($N_{BS}\times N_{UE}$) multiple-input–multiple-output (MIMO) is considered, where $N_{BS}$ and $N_{UE}$ are the number of antennas for the BS and UE, respectively. The power and channel allocation problem is described through a Minimum Cost Network Flow (MCNF) graph model, which is similar to a bipartite graph but with the difference that its vertices can have self-connections (an edge connecting a vertex to itself). These describe the vertices’ parameters (their throughput gain). The graph connects all channels to each of the users, and describes the resulting throughput, which a particular allocation can generate. Through the simplex network algorithm, the optimal allocations are determined, such that every user’s minimum requirement is satisfied. Linear increase in the total throughput $R_{T}$ with the number of users and nearly constant average throughput per user ${\bar{R}}_{u}$ are reported. A scenario in which middle-mile Long Term Evolution (LTE) networks operate in unused TV broadcast frequencies is the basis for the channel allocation problem solved in [20]. A conflict interference graph connects all BSs (vertices) which interfere with each other. The algorithm aims to avoid the allocation of the same channels (provided that they are available for sharing, i.e., unused by the TV broadcast network) to neighboring BSs, subject to a pre-defined value of the fairness index β (dependent on $R_{T}$). The proposed solution provides significantly higher throughput gains; $R_{T}$ declines with the increase in the number of BSs due to the higher interference levels, but the fairness index remains nearly constant. The authors of [21] study RA for an OFDMA small-cell network with prioritization of the users’ data streams. The considered system model includes a varying number of indoor small cells and users served by them, on a variable number of channels. An interference conflict graph is constructed from the UEs as vertices, which are connected with edges if they are associated with an interfering small cell (one that is closer than a certain distance to a user’s serving cell). Furthermore, users are divided into a high- and low-priority sets, which—together with whether a user is associated with a small cell—are also taken into consideration by the channel allocation problem. To reduce the computational complexity, the graph is transformed into a cordial one (if a group of vertices form a closed cycle, then there is at least one chord that connects two vertices on the opposing ends). Then, a heuristic admission control algorithm is employed to associate the UEs with the most appropriate cells, in accordance with the users’ RB demands. Finally, the RBs are allocated. In addition, the authors describe the necessary procedures for the implementation of the proposed scenario in an LTE network. The algorithm’s performance is evaluated through the ratio of guaranteed users $\alpha_{u}$ (sum of satisfied users divided by by their overall number), the throughput satisfaction rate $\theta_{u}$ (ratio of the achievable and required throughputs), and β (which describes how fairly the RBs are distributed). The results show that $\alpha_{u}$ is significantly improved compared to other methods, and remains nearly unchanged as the number of small cells increases. In addition, its complexity is many orders of magnitude smaller. Both $\theta_{u}$ and the fairness index are relatively stable if the number of available RBs is sufficient.

### 4.2. RA Methods with User and Cell Clustering

RA in Het-Nets via clustering of APs and users is explored in [22]. The number of APs, users and channels are fixed, and they are considered to be distributed in rooms on a single floor of a building in this scenario. An interference graph which connects all users is utilized to describe the network, with the edges’ weights being determined as the maximum interference among the two connected users. The users and their serving APs are divided into clusters according to their interference vertices (users), with the minimum degree between themselves forming a cluster. The algorithm aims to choose channels and power which ensure that the intra-cluster interference (between the edges of the users within a cluster) is minimized. Notable performance gains are reported for both the ${\bar{R}}_{u}$ and the average throughput per cell ${\bar{R}}_{c}$. This problem is further expanded by considering User-Pair Resource Allocation, i.e., users served by different APs are grouped together so RA is performed on clusters instead of individual UEs [23]. The optimization problem is decomposed into multiple minimum path selection sub-problems which are formed by clusters of varying sizes, such that the weighted sum-rate (WSR) of each user-pair is maximized. The WSR is comprised of the throughputs of the pair and arbitrarily weighted according to the quality-of-service (QoS) requirements of each user. These weights are updated after each RA instance. Similar to [22], the clusters are formed by intra-path sum-weights, i.e., the sum of all edges weights between the vertices within a path. User-centric (UC) clustering in a cellular network, comprised of a fixed number of UEs moving in an area covered by a variable number of BSs, is used as the scenario for which RA with overhead minimization is studied in [24]. The particular aim of this work is to allocate channels for the overhead needed for the formation of user clusters. The graph’s vertices describe the BSs, their color illustrates the channel which they utilize (in time domain), while the edges represent conflict in the channel allocations (i.e. the critical interference threshold is exceeded) between two BSs. Then, the problem is solved by arranging the BSs such that maximal orthogonality between the channels allocated to them is achieved. BSs and UEs are clustered on the basis of the channel gain between them. The results show that $R_{T}$ increases linearly with the number of BSs and channels per BS, as well as with the cell radius. A multiple connectivity (MC) cellular scenario, characterized by the association of one UE to multiple BSs, is used as a basis for the RA method in [25]. The scenario includes multiple small cells and UEs, to be allocated a fixed set of channels, and distributed within the coverage area of a single-macro BS. Additionally, downlink/uplink (DL/UL) decoupling is applied for some UEs, i.e., they associate with different BSs for their DL and UL connections. An interference graph is constructed for user clustering. The set of UEs comprises the vertices, all of them are connected via edges, and the sum of their DL and UL interference describes the edges’ weights. The users are grouped in clusters according to their interference, so that the sum of the weights in any cluster is maximized. To distribute the resource blocks (RBs) among the users, a bipartite graph is formed by the sets of user clusters and RBs (they are of the same number), with the achievable throughput for each RB–user pair describing the edges’ weights. Linear throughput gain with the increase in the number of small cells is reported. User grouping for RA in a non-orthogonal multiple access (NOMA) cellular network that includes a single macro BS serving a variable number of UEs is proposed in [26]. The power consumption and externality (PCE) function is introduced to characterize a user’s power consumption, as well as the interference that the user creates toward the others in a group. The channels assigned to each group are assumed to be orthogonal, and the algorithm’s task is to allocate power to each UE in the group in accordance with its channel quality and PCE. A directed graph (pointing from the user with the highest channel gain to that with the lowest) is constructed, which includes all users, including a set of virtual users (each representing a user group), with the edges connecting only users in different groups. The edges’ weights illustrate the PCE differences between two vertices. Power allocation is performed via a fast greedy algorithm that aims to find the minimum total edge weight between every two vertices. The solution provides fast convergence and significantly lower total network transmit power $P_{T}$ than the alternatives. In a similar manner, the authors of [27] group the users in clusters, allocating orthogonal sets of RBs for each cluster (then, only the intra-cluster interference needs to be considered), and then solve the joint RA and energy efficiency (EE) problem for a NOMA system. Their focus is on matching the users to the RBs via a many-to-one bipartite graph i.e., each user is matched to a single RB, while each RB matches with $L_{u}$ users which operate through NOMA ($L_{u}=\left\lceil N_{UE}/N_{RB}\right\rceil$, where ⌈.⌉ indicates the ceiling and $N_{RB}$ is the overall number or RBs). The UEs swap their RBs until maximum EE is achieved for each cluster (power is assumed to be known). Then, for each RB, a power allocation which aims to maximize the throughput with the constraint of the maximum transmission power is performed. This problem is solved analytically for the general case of two users per RB (i.e., just two transmission power levels), for variable numbers of UEs, RBs and maximum power levels, achieving decreased computational complexity. Convergence of the EE ε is reached at $P_{max}=-5$ dBm.

### 4.3. RA Methods with Belief Propagation

The RA problem with a focus on ICI coordination is considered in [28] for a Het-Net that includes a fixed number of UEs and channels and a variable number of BSs. A factor graph is formed, with its variable vertices illustrating the optimization variables (each optimized by a separate BS), while the factor vertices are utility sub-functions (which together form the RA optimization problem), and their number is equal to $N_{BS}+N_{UE}$. Edges between the factor and variable vertices exist only if there is an opportunity for connection between the BS and UE, which are related to a particular factor and variable vertices. Then, the utility sub-functions are individually approximated (ICI is modeled as a Gaussian function) through belief propagation (BP) among the neighboring factor vertices. Due to the Gaussian approximation, each vertex needs to only propagate the mean and variance of the utility sub-function, which increases the computational efficiency. This method adapts the small cells’ transmission power according to their density, to reduce the ICI. Q-learning is used in [29] to implement computationally efficient RA for a millimeter-wave (mmWave) Het-Net that encompasses a single macro BS and a fixed number of UEs, small cells, and channels. The time-averaged risk-averse rate AR (dependent on the throughput) of a particular BS-UE link is used to define the utility function. At each instance, a coordination graph between neighboring BSs and UEs is constructed to reflect the associations of UEs with BSs, as well as the interference between BSs. Then, through BP of the Q-function (dependent on the AR) among neighboring vertices, the RA is performed by the BSs in a distributed manner. If a node’s AR is greater than a threshold, then its state changes and it obtains a new association and RA; otherwise, it is not included in the estimation process. Further computational speed is achieved by taking into account the past states of the Q-function. The simulations are performed with the use of real-world measurements, showing quick convergence of the learning algorithm and a linear dependency of the throughput with the BS transmission power.

### 4.4. RA Methods Based on GSP

The authors in [30] explore the application of GNNs for modeling cellular networks and solving popular problems for them. A general variant for graph construction is adopted—BSs and UEs are the vertices, connected via edges if there is an association between a BS and a UE, or two BSs interfere with each other’s transmissions. Edges are not drawn between nodes which are over a certain distance apart from each other because their signals’ strengths are neglected. The edges’ weights are represented as the channel gains for each link. At a particular vertex, information from its neighbors is aggregated, and then combined via convolution to extract the graph’s topological features. The Adam optimization algorithm [41] is applied for the GNN training procedure. It converges in a few iterations, and the throughput results remain stable. The GNN for Het-Nets is further adapted for learning optimal power allocation when the adjacency matrix does not exhibit universal permutation equivalence and invariance [31]. The authors propose a more advanced user clustering by dividing the UEs into $N_{BS}$ subsets (all users served by the same BS compose one subset) and processing them separately through the GNN’s layers. To achieve this, the GNN’s weight matrix is formed by $N_{BS}\times N_{BS}$ sub-matrices, which process the information of each UE subset. The bipartite graph is therefore composed of both the BSs and the UEs as vertices (all BSs have $P_{max}$ as a vertex attribute), with their associated and interference links being the edges (their attribute is the channel gain). The RMSProp [42] optimization algorithm is applied. Substantial computational efficiency for the scenario with fixed numbers of UEs, macro BSs and small-cells, in comparison with the alternatives, has been achieved. A novel GNN architecture via algorithm unfolding for power allocation in an ad hoc network is proposed in [32]. The objective function of the throughput is decomposed into several parameters which process the vertices’ information iteratively, and are dependent on the channel gain (which is the vertex attribute). Every connection between a transmitter and a receiver comprises a vertex, with the edges being the interfering connections. Optimization is performed through stochastic gradient descent (SGD). A significant reduction in the computational complexity compared to the alternatives is reported. An alternative solution to the power allocation in ad hoc networks is proposed in [33,43]. The RA policy (i.e., the processing layer’s parameters) is trained for the whole graph, whereas it is implemented locally (for each transmitter–receiver pair). The graph is composed of the transmitters as vertices, and the interfering links between them as edges (the channel gains being their weights/attributes). For each vertex, the information from its immediate neighbors is aggregated to capture the asynchronism of the interference, or the synchronism of the associated communication links. The result is then filtered through the GNN’s layers to obtain the power allocations. The layers’ parameters are obtained via alternating the gradients between the variables of an Lagrangian function (through a convolutional neural network) dependent on the channel gain, and the adjacency matrices. Asynchronous SGD is utilized to train the model. Close to linear gain in ${\bar{R}}_{u}$ occurs with the increase in the number of transmitter–receiver pairs in the testing stage. Further developments are made by the DRL framework for channel allocation, based on GNNs in wireless local area networks (WLANs) with reduction in the number of states, as proposed in [34]. The graph is formed from the APs, and all of them that are able to detect each other’s signals are connected via edges. At each instance, the Q-learning processes each AP’s current state via the GNN and chooses the channel allocations (from the replay buffer of stored policies), such that the reward (a function of the throughput) is maximized. The number of APs, UEs and channels are fixed in the considered scenario. The usage of replay buffer has been shown to significantly decrease the effect of over-fitting for the GNN. Fast convergence is also indicated.

### 4.5. Miscellaneous Methods

RA and EE with distributed optimization in a Het-Net comprising a fixed number of macro BSs and small BSs (or APs) is studied in [35]. The proposed solution utilizes a Gibbs sampler (describing the SINR of each user) operating on a graph to minimize the interference (and hence optimize the throughput) between a macro BS and a small BS. The users represent the graph’s vertices, whereas a user’s neighbors receive a signal stronger than a certain threshold (i.e., interference) on the same channel, thus constituting an interference graph. Each vertex has an attribute describing its power and channel allocation, determined by its serving BS. Substantial ${\bar{R}}_{u}$ and energy efficiency ε gains are achieved. Channel distribution according to the users’ location (close to the cell’s center or its edge) in the scenario of fixed number of BSs, UEs, and channels, is performed in [36]. The BSs that serve users with unfavorable conditions (DL SINR below a certain threshold), are described as vertices of the graph, while the edges connecting them illustrate the interference levels. The optimization problem aims to maximize the center users’ throughput while limiting the BS transmission power allocated to them, and provide orthogonal channels to the cell-edge users. An alternative approach to solving this RA problem is proposed in [37] by dividing it into two sub-problems. Firstly, UA and channel allocations are performed for fixed power from the BSs to the UEs. A bipartite graph is used to describe the connections between them. The channel allocations are performed via the Hungarian algorithm, which aims to match the BSs to the UEs that produce the maximum weighted sum throughput (MC is considered). Then, the power allocations are performed in a separate optimization problem that utilizes the already obtained channel allocations. It is solved via the difference of the convex functions approximations (DCA) method. Finally, the two methods are combined to obtain faster convergence. This study is extended in [38] by considering the co-tier (between the cells of the same type) and cross-tier (cells of different types) interference. The system’s resources are divided into regular resource blocks (RRBs) and almost bank resource blocks (ABRBs), the latter being allocated to reduce the cross-tier interference, while allocating orthogonal RBs to neighboring cells aims to deal with the co-tier interference. The two tiers (small cell and macro-cell) are described via two separate interference graphs. Vertices are BSs of the same tier, and if the distance between two of them is greater than a threshold, they are connected with an edge. Afterwards, these graphs are converted to conflict graphs which are composed of the cells’ UEs, and there are edges between those served by cells that were connected together in the interference graphs. Then, the groups of two or more distinct adjacent vertices (cliques) form trees that are used for RB allocation in the cells of each of the tiers. The final RB distribution is performed via an exhaustive search algorithm, with the minimum throughput satisfaction rate (TSR) constraint. This solution achieves a relatively stable ratio of ABRB, of $\theta_{u}$, and of β as the number of small cells increases. Predictive ICI and RA in Het-Nets with spatial division multiple access for a fixed number of BSs and variable number of UEs is proposed in [39]. The DL data stream is divided into frames, and the algorithm determines which BS is to serve which user at each frame (interfering BSs do not transmit for its duration). In addition, each BS has multiple antennas that serve different users, and in this way, each antenna forms a virtual BS. The users and virtual BSs are described as the vertices, whereas their connections—via the edges, their weights being the network utility parameter—are dependent on the TSR $\theta_{u}$. Then, the graph’s vertices are divided into independent sets (a subset such that no two vertices in it are connected). The channel allocations which provide the highest utility (maximum weight) for the users in each independent set, are then found.

### 4.6. Lessons Learned and Trends in Development

The summary presented in this section shows several features of the research in graph-based RA algorithms for homogeneous and Het-Nets, which provide hints for their further development. They are as follows:

- In this deployment scenario, the most prominent problem is RA because it is usually assumed that: (1) UA is performed independently through traditional methods, and (2) there is no inter-network spectrum sharing (SSh), i.e., the spectrum access is static, which is the usual case for this type of network. Due to the cell-edge throughput degradation problem, however, UA and RA should be considered together, and graph-based algorithms are a potent solution [28,29,37]. *A significant challenge of designing them lies in how to properly group the different sets of vertices (UEs, macro cells, small cells, and APs), so that their constraints on the transmission power may be satisfied. In addition, the users in each tier are influenced by interference in an non-reciprocal manner (a small-cell user may receive more interference from a nearby macro cell than a user served by that cell).* This also needs to be considered in the way that the graph’s edges are defined. Furthermore, the optimization problems will involve multiple independent variables such as the channel gains, power allocations, available channels, and distance between the nodes.
- When it comes to graph-based RA algorithms for Het-Nets, little attention has been given to the influence of the channel’s impairments (such as UE movement speed, fading, and noise distributions) on the received SINR, and thus to the throughput. *Consequently, there is worth in considering whether a particular graph topology can take advantage of the dynamic network conditions (for example, the graph’s edges may need to be readjusted often with the movement of the UE). Modeling the Het-Net via GNN and considering these conditions as attributes of the vertices and edges can provide effective solutions in this case.* These challenges become even more intricate when it comes to whether a particular set of channels is available or not, i.e., when multiple radio access technologies (RATs) coexist in the same or adjacent portions of the spectrum [20]. Then, dynamic and intelligent multiple access needs to be implemented through CR methods (discussed in Section 6).
- Several noteworthy works [44,45,46,47,48,49] have expanded the scope of RA methods for communications between UAV and satellite nodes, as well as their connection to terrestrial Het-Nets. The presented graph models are hereby briefly reviewed, and the relevant challenges described. Reliable connectivity between satellite nodes, considering the temporal changes due to their varying movement velocities, is considered in [44,45,46,47]. The creation of edges is determined by the duration of direct visibility between two communication nodes and their link latency. Network topology is adapted in time by the vertices’ attributes, according to the shortest path length and relevance (number of shortest paths going through a vertex) [44]. Alternatively, an RA solution is proposed in [45] which constructs the time-varying graph using two types of edges. They represent caching links and communication links, with each type of edge connecting two vertices (satellites/high-altitude platforms) that are related by either the cached data at one of the nodes, or by their information exchange. This approach is built upon in [46] by a commodity-flow algorithm for frequency scheduling of ground-based, UAV, and satellite nodes, and the links between those that communicate directly for the provision of a particular service. In [47], both the change in the nodes’ available bandwidth and their traffic load are modeled by a Markov model, jointly with the time-varying graph that models the space and terrestrial nodes’ movements. The connections between these nodes (vertices of the time-varying graph) constitute the edges, which change with the fluctuation of link availability (i.e., when handover occurs). This dynamic is described by the angular parameters of the satellites’ positions. RA is performed separately for the ground and space-based nodes. Link establishment that satisfies the shortest path in a graph for terrestrial, aerial and space communications is explored in [48]. The processing power and link delay between a node and the ones connected to it are considered, and they define the vertex attributes. RA is determined for specific user requirements modeled by a utility function. User pairing for NOMA has been modeled in [49] by a bipartite graph for terrestrial mobile users, the coverage of which is supplemented by a satellite node when they are outside of BS range. The channel gain between the users defines the edge attributes, and the pairing is solved via maximal matching to increase the throughput of the terrestrial BSs. *The limitation of accumulating graph topology changes in time; however, it comes from the number of vertices and connections between them, as larger graphs will require not only a more significant computational burden on each iteration, but also a more complex accumulation process.* Further development can be achieved through distributed topology generation via federated learning (FL) [50]. In addition, the implementation of the overall RA algorithm often includes several sub-problems (channel and power allocation, user grouping, handover, multiple access, etc.) that are solved independently [37,40,49]. Then, *the challenge is to define and solve each of them in the most computationally efficient manner using traditional optimization methods or deep learning, with respect to the essential parameters (such as the number of channels and communication nodes).*
- Considering the advantage of using graph-based methods to describe the physical characteristics of the communication nodes of homogeneous and Het-Nets, their relationships, and the disadvantage of computational complexity due to allocation of many users, channels, and power levels, the review in this Section has shown that GNNs are promising for decreasing the computational load of standard heuristic optimization solutions. However, their implementation requires the following considerations: *(1) the training method involves either a preliminary simulation of the network’s operation to obtain data about the nodes and their relationships (in supervised learning), or the design of a RL procedure; (2) it involves definition of the relationships between the network’s nodes as components of the graph; (3) addressing MC (one UE being connected to multiple cells) and UA requires differentiation between the vertices that describe BSs and UEs; (4) attributes (such as interference levels, channel gain, associated nodes) of the vertices and edges may facilitate the learning of more complex graphs.*
- Most graph-based methods in this category are evaluated for the DL case, but they may not be naturally applicable for UL communications because the interference sources (i.e., other UEs) are much greater in number than for the BSs (in the cellular Het-Net scenario). *Their transmission power is also much weaker, which needs to be considered when determining the criterion (i.e., distance) of drawing an edge between two interfering users.*

## 5. Graph-Based RA in D2D Networks

**Figure 5:**
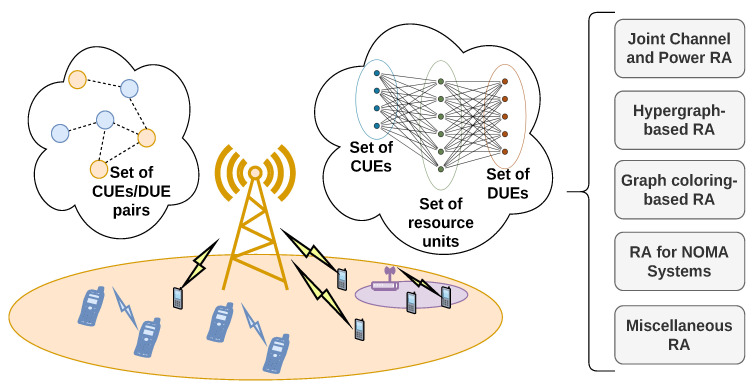
Scenario of D2D networks and summary of methods for graph-based RA.

D2D networks have created a promising paradigm for the integration of distributed close-range communications within the range of other networks (mostly cellular networks). These applications include vehicular communications, IoT-based services, and cellular offloading (users that request the same content can have it shared by others in their vicinity, rather than downloading it separately through the BS) [6]. Generally, the D2D nodes are considered in pairs (a transmitter and receiver), while the cellular users are considered individually. Even though D2Ds operate in both inband (in the cellular band) and outband (in unlicensed bands) modes, graph-based RA has been utilized mostly in the former mode of deployment, via underlay spectrum access (the D2Ds utilize the spectrum opportunistically, so that they may not impede the cellular communications). This is because RA in the outband mode may lead to overly complex graph topology due to the inclusion of an additional set of frequency channels, and the difficulty in overhead exchange between the D2Ds and the BS. The reviewed methods aim to minimize both interference from the UL communications of cellular UEs (CUEs)/DL transmissions of the BS to the D2D pairs, as well as that resulting from their own exchange towards other D2Ds, the CUEs, and the BS. Most of these works use a system model comprised of a macro-cell BS with $N_{CUE}$ CUEs and $N_{DUE}$ D2D pairs (DUEs) operating in its range. The scenario of D2D networks is illustrated in Figure 5. A generalized illustration of the most common graph models for RA problems (usually used independently from each other), and a summary of their subcategories comprises this Section. A tabular summary of the main design characteristics of the algorithms reviewed in this Section is given in Table 7.

### 5.1. Joint Power and Channel Allocation Methods

A joint power and channel allocation method for the scenario of a variable number of CUEs and DUEs in the coverage of a single macro BS is developed in [51]. It allows for multiple RBs to be allocated for a single (D2D or cellular) communication link, and for an RB to be utilized by different links. To this end, an interference graph is employed, its vertices representing the CUEs and DUEs, while its edges represent their interference relationships (for each edge, the weight is a vector of interference levels on all RBs). Each vertex has attributes that characterize their current RB and power allocation, as well as their desired RBs. The algorithm forms clusters of CUEs and DUEs that use the same RBs, such that the cluster’s throughput is maximized. It has been found that this solution is scalable due to its weak dependence on the number of vertices, while $R_{T}$ increases. The authors of [52] also study the joint power and channel allocation problem in a similar scenario, with the aim of achieving the minimum throughput requirements of both CUEs and DUEs. First, the power allocation is performed separately under the consideration that each node can only occupy a single channel. Then, through a tripartite graph connecting the channels, CUEs, and DUEs, the channel distribution is chosen so as to maximize the edges’ weights (achievable throughput per allocation) for both the CUEs and DUEs. This is performed by applying the Hungarian algorithm on a tree topology that represents all possible channel allocations via its branches. The results indicate that due to the limitation of each link to only utilize a single channel, there is a critical number of CUEs, which if exceeded, the DUEs’ outage will increase. Nevertheless, the proposed method prevents a dramatic decline in $R_{T}$. Further, a joint power and channel allocation for underlay D2D communications, subject to the constraints of CUEs’ throughput and secrecy data rate (the fraction of the data rate that has not been tampered with by a malicious D2D node), is developed in [53] to improve not only RA, but also the resilience to physical layer attacks from unauthorized DUEs. First, power allocation for all nodes is performed so as to satisfy the CUEs’ throughput requirements. Then, the channels are allocated through a bipartite graph connecting the CUEs and D2D pairs, with the edges’ weights representing the achievable SINR of the DUEs, if the CUEs’ channels are shared. An exponential increase in the $R_{T}$ with the number of both DUEs and CUEs, is reported.

### 5.2. Hypergraph-Based RA Methods

A more thorough interference coordination for RA in underlay inband D2D communication for a variable number of CUEs and DUEs within the coverage of a single macro BS is performed in [54] using a hypergraph-based method. It is a generalization of the standard graph, in which the hyperedges connect any subsets of the vertices, rather than just two, and its topology is reflected in the incidence matrix only. The CUEs and DUEs constitute the hypergraph’s vertices, while their interference defines the hyperedges depending on whether the individual nodes or multiple users cumulatively introduce interference greater than a predefined SINR threshold. As determined empirically, a hyperedge may encompass, at most, two other vertices apart from the one considered (increasing the hyperedge’s scope does not yield meaningful gains). Channels are allocated through graph coloring, with the limitation that within one hyperedge, only one channel may be used for each vertex. The proposed solution achieves near optimal results, as $R_{T}$ increases with both $N_{CUE}$ and $N_{DUE}$. An alternative vehicular communications scenario is considered for the RA problem in [55], with the goal of providing channel allocation between a fixed number of vehicle-to-infrastructure (V2I) and vehicle-to-vehicle (V2V) nodes. The former communicate with the BS (their links are prioritized), while the latter communicate with each other (their links are subject to optimization). V2V links are clustered according to their interference, operating on one RB allocated for a V2I link, while neighboring clusters have to use different RBs. A tripartite hypergraph is composed of the RBs, V2I and V2V nodes as vertices, with their edges’ weights corresponding to the achievable throughput. Each cluster of V2Vs is considered in a separate hyperedge connected to the RBs and V2I vertices. Power allocation is performed separately. The proposed solution shows increased V2I total throughput for the high SINR levels at the V2Vs, and so exhibits resilience in fast fading. The study [56] explores a NOMA-based vehicular communications scenario with channel allocation between V2Is, broadcast V2Vs (a group of V2Vs where one of them transmits messages to all the others), and standard V2Vs, each of these types forming separate communication groups (the latter two being underlay users of the V2I spectrum). Two kinds of interference are considered—between each group, and within the groups themselves. The broadcast V2Vs and standard V2Vs are divided into clusters (using the Hungarian algorithm) based on their mutual interference. Then a tripartite hypergraph is constructed, which has the clusters, V2Is and RBs (they are all of an equal number) as its vertices, and the matching relationships between them as hyperedges (their weights being the achievable throughput for each allocation). The algorithm aims to maximize their weights through either a greedy or an iterative algorithm, which both exhibit similar performance, but the former has lower complexity. Simulation results show exponential gain in $R_{T}$ with the increase in the number of nodes, as well as a decline in performance as the vehicles’ speeds increase. The authors of [57] consider the channel allocation between CUEs, V2V, and V2I nodes (all operating within the range of a single macro BS), such that the probability of outage requirements for V2Vs and the SINR requirement of the cellular users are met, while the throughput of V2Is is maximized. Power allocation is performed separately via the Newton method. A hypergraph is constructed similarly to [58], with all three types of users being represented as vertices, while their edges describe the interference from individual neighboring nodes, and hyperedges denote the cumulative interferers for each vertex. Then, the RB allocation is performed through graph coloring on the condition that two vertices connected by an edge cannot share the same RB. This is performed via a three-dimensional adjacency matrix (each user type being a separate dimension) that is adjusted in each iteration.

**Table 7:** Tabular summary for graph-based RA in D2D networks.

Reference	Application	Graph Model Type	Tasks Solved via Graphs	Graph Formulation	Optimization Method	Performance Assessment
[51]	Channel allocation for CUEs and DUEs	Interference graph	Channel and power allocation	Vertices—CUEs and DUEs; edges—between CUEs and DUEs	Greedy algorithm	$R_{T}$ of 2.4 Mb/s for $N_{CUE}+N_{DUE}=35$
[52]	Channel allocation for CUEs and DUEs	Tripartite/tree graph	Channel allocation	Vertices—channels, CUEs, DUEs; edges—between channels, CUEs, and DUEs; weights—throughput	Hungarian algorithm	$R_{T}$ of up to 650 b/s/Hz for $N_{CUE}=10$ and $N_{DUE}=48$
[53]	Channel allocation for CUEs and DUEs	Bipartite graph	Channel allocation	Vertices—CUEs and D2D pairs; edges—between CUEs and D2D pairs; weights—SINR	Hungarian algorithm	$R_{T}$ up to 16 b/s/Hz for $N_{CUE}=6$ and $N_{DUE}=20$
[54]	Channel allocation for CUEs and DUEs	Hypergraph	Channel allocation	Vertices—CUEs and DUEs; hyperedges—encompassing CUEs and DUEs, depending on the SINR	Greedy algorithm	Stable $R_{T}$ of up to 600 b/s/Hz for $N_{CUE}=30$ and $N_{DUE}=30$
[55]	RA for V2Vs	Tripartite hypergraph	Channel allocation	Vertices—channels, V2Is, V2Vs; edges—between channels, V2Is, and V2Vs; weights—throughput	Heuristic algorithm	$R_{T}$ of up to 120 b/s/Hz
[56]	Channel allocation for V2Is and V2Vs	Tripartite hypergraph	Channel allocation	Vertices—V2Is, V2Vs, RBs; hyperedges—connecting RBs, V2Is and clusters of V2Vs	Greedy/iterative algorithm	$R_{T}$ of up to 26 b/s/Hz for up to 70 nodes
[57]	Channel allocation for CUEs, V2Is and V2Vs	Interference hypergraph	Channel allocation	Vertices—CUEs, V2Is, V2Vs; hyperedges—encompassing CUEs, V2Is, and V2Vs, depending on the SINR	Greedy algorithm	$R_{T}$ of up to 105 b/s/Hz for $N_{CUE}=20$ and $N_{DUE}=40$
[59]	RA for DUEs	Interference graph	Channel allocation	Vertices - D2D pairs; edges—between the D2D pairs; weights—interference	Heuristic algorithm	$R_{u}$ of up to 1 Gb/s
[60]	Channel allocation for FD CUEs and D2Ds	Interference graph	Channel and power allocation	Vertices—DL CUEs, UL CUEs, DUEs; edges—between DL CUEs, UL CUEs, DUEs; weights—interference	Heuristic algorithm	$R_{T}$ of up to 38 b/s/Hz and β=0.5 for 120 links
[58]	Channel allocation for CUEs and DUEs	Bidirected interference graph	Channel allocation	Vertices—CUEs, DUEs; edges—between CUEs and DUEs; weights—cumulative interference	Tabu search algorithm	$R_{T}$ of up to 410 b/s/Hz and β=0.85 for $N_{DUE}=100$, $N_{CUE}=10$
[61]	Channel allocation for CUEs and DUEs	Interference graph	Channel allocation	Vertices—D2D pairs; edges—connecting the interfering D2D pairs	Heuristic algorithm	Average DUE satisfaction up to 70% for $N_{CUE}=10$ and $N_{DUE}=120$
[62]	Channel allocation for CUEs and DUEs	Bipartite graph	Channel allocation	Vertices—CUEs, DUEs; edges—between CUEs and DUEs; weights—EE	Hungarian algorithm	ε of up to 1.5 kb/s/Hz for $N_{DUE}=15$, $N_{CUE}=10$
[63]	Channel allocation for CUEs and DUEs	Bipartite graph	Channel allocation	Vertices—D2D pairs and channels; edges—connecting D2D pairs and channels; weights—throughput	Hungarian algorithm	$R_{T}$ of up to 95 b/s/Hz for $N_{CUE}=6$ and $N_{DUE}=16$
[64]	Channel allocation for CUEs and DUEs	Interference/ bipartite graph	Channel allocation	Vertices—CUEs/DUEs/CUE and DUE clusters; edges—connecting all respective vertices; weights—channel correlations/interference	Heuristic/ Hungarian algorithm	$R_{T}$ up to 100 b/s/Hz for $N_{CUE}80$ $N_{DUE}=80$
[65]	Channel allocation for CUEs and DUEs	Bipartite graph	Channel allocation	Vertices—CUEs and DUEs; edges—between CUEs and DUEs	Hungarian algorithm	$P_{out}<0.1$ at $\gamma_{T,DUE}>25$ dB and $\gamma_{T,CUE}<22$ dB for $N_{CUE}=30$ and $N_{DUE}=30$
[66]	Channel allocation for CUEs and DUEs with social relationships	Interference/ social graph	Channel allocation	Vertices—CUEs and DUEs; edges—between CUEs and DUEs	Potential game	$R_{T}$ of 11 b/s/Hz for $N_{CUE}=15$ and $N_{DUE}=55$
[67]	Channel allocation for CUEs and DUEs with limited CSI	Bipartite/ interference graph	Channel allocation	Vertices—CUEs and channels/CUEs and DUEs; edges’ weights—received signal/throughput	Heuristic algorithm	Stable $\Omega_{U}$ for $N_{CUE}=5$ and $N_{DUE}=12$
[68]	Channel allocation for CUEs and DUEs	Bipartite graph	Channel allocation	Vertices—channels, DUEs; edges—between channels and DUEs; weights—PF	Heuristic algorithm	$R_{T}$ of up to 12 b/s/Hz and β=0.8 for $N_{CUE}=20$ and $N_{DUE}=20$
[69]	Channel allocation for CUEs and DUEs	Interference graph	Channel allocation	Vertices—small cells; edges—between interfering small cells	Hungarian algorithm	$R_{T}$ of up to 5 Gb/s for $N_{DUE}=N_{CUE}=500$
[70]	Channel allocation for CUEs and DUEs	Interference graph	Channel allocation	Vertices—CUEs and DUEs; edges—connecting the interfering nodes	Heuristic algorithm	${\bar{R}}_{u}$ up to 2.5 Mb/s and β=0.95 for $N_{CUE}=25$ and $N_{DUE}=150$

### 5.3. Graph Coloring-Based RA Methods

RA in D2D-based information relaying in a mmWave vehicular scenario (the sub-6 GHz spectrum is used for overhead communications with small-cell BSs) is explored in [59]. Each D2D pair constitutes a vertex, and the potential interference between two links forms an edge. Channel allocation is performed through graph coloring, ensuring that all neighboring links operate on separate channels. Choosing a color (channel) is also facilitated by the DUEs sharing information about the interference among themselves. The BSs also receive feedback from all nodes to allocate independent channels for overhead exchange between the users. Relatively stable high throughput, with greater gains for shorter inter-BS difference, has been reported. The authors of [60] explore RA in underlay D2D communications based on full-duplex (FD) transceivers, i.e., they are equipped with separate radio frequency circuits for transmission/reception, and can then use the same channels for both UL and DL. Hence, multiple RBs may be allocated to each link, and a RB may be assigned to different links simultaneously. The scenario for which the RA solution is applied is composed of a variable number of DUEs and CUEs within the coverage of a single macro BS. The most significant challenge in FD communications is the resulting self-interference between the transmitting and receiving antennas. An interference graph illustrates the network as follows: the CUEs DL and UL links are considered as separate sets and, together with the DUEs, are denoted as vertices. They are all connected via edges, the weights of which are the interference levels. Vertices have four attributes—type (UL/DL CUE, DUE), vector of the received signal powers on each RB, vectors of colors that show which RBs are allocated to this vertex, and vectors of permitted transmission powers for each RB. The coloring is performed on the basis of the achievable throughput and the interference for a group of neighboring vertices. Simulations show fast convergence, a linear increase in $R_{T}$ with the number of nodes, and stable fairness. The proposed algorithm is highly dependent on the self-interference index, which is subject to the transceiver’s design. An alternative graph structure [58] that considers non-adjacent interference sources is devised for a D2D-based IoT network composed of a variable number of CUEs and DUEs, with a fixed number of channels. A bidirected graph is composed of the CUEs and DUEs as vertices, and the interference between every pair of them is illustrated via edges. The graph coloring is performed by first assigning unique channels to the CUEs, followed by allocating resources for each DUE, such that its potential interference to other unassigned DUEs may be minimal (the interference level at each edge includes the cumulative unwanted signal power of all other vertices in the graph). The algorithm aims to distribute the channels such that the distance between two interfering nodes may be maximized. For greater efficiency, the tabu search heuristic algorithm [71] is used to obtain the solutions. It achieves significant gains for $R_{T}$ as $N_{DUE}$ increases, even though its convergence speed decreases significantly. In addition, notable improvement of the fairness index in comparison to other graph coloring methods has been reported. A graph-based user clustering of D2Ds under the constraint of the outage probability of the CUEs is proposed in [61]. The channels are allocated preliminarily for the CUEs, and are orthogonal to each other. Each D2D pair constitutes a vertex in the graph, and there is an edge between two vertices if their achievable throughput is lower than a threshold. Channels represent colors, and are allocated through graph coloring so as to maximize the DUEs’ satisfaction function, which is dependent on the DUEs’ throughput requirements, their actual data rate, and on the number of neighboring vertices that use the same channel (it should be minimized). All D2D pairs that do not utilize duplicate channels form a cluster. The algorithm uses limited channel state information (CSI) to reduce the overhead. Simulation results show that CUEs outage probability stays minimal with the increase in the DUEs’ number, while the satisfaction function is maximized when the interference tolerance among the DUEs is equal.

### 5.4. RA Methods in NOMA Systems

RA in NOMA-based D2D communications is explored in [62]. The system model involves CUEs (each utilizing a single channel orthogonal to the others), as well as D2D groups composed of a transmitting node and multiple receiving ones (operating on a single CUE channel through NOMA). The channel allocation problem is described via a bipartite graph which connects the D2D groups (each represented by its transmitting DUE) and CUEs, the edge’s weights being the EE ε. Then, the optimal allocations (channel sharing between a CUE and a D2D group) are found via the Hungarian algorithm. Afterwards, power allocation is performed via a separate KKT-based method. The proposed solution exhibits a linear increase in the EE with the number of D2D groups; however, it gradually declines as the D2D transmission power is increased. To improve the interference resilience of underlay D2Ds for NOMA, the authors in [63] propose the interlay spectrum access mode (user multiplexing in the power domain). Thus, based on the interference levels, the proposed solution chooses which of the two modes to assign for a link, together with the power and channel allocation. The graph’s vertices represent all possible combinations of underlay D2D pairs, interlay D2D pairs, and their allocated channels. Two vertices are connected via an edge if they do not include the same D2D pairs and the same channel allocations. It is the vertices, rather than the edges, that have a weight attribute, which represents the maximum throughput achieved by the combination. Then, the vertices with maximum weights are determined through a pruning algorithm, and in this way, the channel allocations for the D2Ds operating in each of the modes are generated. Afterwards, the optimal power for each vertex is determined via an iterative algorithm. To relieve the computational burden of these operations, a bipartite graph connects the D2D pairs and channels, with the throughput for each allocation being the edge’s weight. Through the Hungarian algorithm, the allocation is performed with consideration of the choice of mode for each D2D pair that yields less interference. Exponential gain in $R_{T}$ is reported with the increase in the number of D2Ds. It is also retained when the CUEs’ transmission power increases. Underlay D2D for a mmWave MIMO-NOMA system is considered a model for which user clustering and power allocation solution is employed in [64]. It involves a macro cell, and within its range operate small cells that serve clusters of CUEs with orthogonal channels being assigned to each cluster, while the users in it use NOMA. The underlay D2Ds within each cluster utilize the same channels as the CUEs, aiming to maximize the network throughput while guaranteeing the QoS of both the DUEs and CUEs. The CUE clustering is performed through a graph with the CUEs as vertices that are connected by edges, the weights of which represent the channel correlations between each two. Then, the vertices are clustered through *K*-means on the basis of the correlations between them. The DUE clustering is performed through a graph with the D2Ds as vertices connected by edges (interference). Finally, the two sets of clusters are matched through a bipartite graph, with the edges’ weights being the channel correlations between each CUE and DUE cluster. The power allocation is performed separately via a particle swarm optimization algorithm [72]. Through simulations, it is discovered that the proposed solution achieves fast convergence, as well as the optimal cluster sizes and number of DUEs.

### 5.5. Miscellaneous Methods

The authors of [65] propose distance-dependent RA (which does not require the users’ channel state information, or CSI, at the BS) through two separate approaches for minimization of the outage probability $P^{out}$ (likelihood of not reaching a certain critical SNR at some D2D receiver). To describe the optimization problems, they are modeled via a bipartite graph, the vertices of which are the D2D pairs and CUEs, while its edges are weighted by the $P^{out}$ for each connection. Both a shortest path (finding the one with the lowest outage) algorithm and binary search (choosing the topology that yields $P^{out}$ under a predefined threshold) are used to determine how channels are to be allocated for the D2D pairs, considering that they are all used by the CUEs. Simulations show that $P^{out}$ increases exponentially with the target SINR $\gamma_{T,CUE}$ of the CUEs, and $N_{CUE}$, while it decreases linearly with the increase in $N_{DUE}$, and their target SINR $\gamma_{T,DUE}$. Introducing social relationships between the mobile users into the graph-based RA for D2D and cellular coexistence (i.e., considering both the social and physical domains) has been explored in [66]. Thus, social and an interference graphs are constructed, which both include the CUEs and DUEs as vertices, while their edges describe the closeness coefficient which denotes their social relationships (in the former graph) or interference levels (in the latter graph). Then, the problem is formed as optimization of the social utility function $U_{s}$ that is composed of the CUE and DUE throughputs, and their closeness, under the constraint that a D2D pair employs only one RB. A Nash equilibrium potential game is defined to perform the channel allocation. Simulations have shown that $U_{s}$ increases with the number of DUEs, whereas the fairness exhibits a relative decline. Furthermore, increasing the social link probability yields throughput gains because it means that more D2Ds are eligible to share common content and offload traffic from the BS. Channel and power allocation in the condition of limited CSI (geographical locations of DUEs, and CSI from the CUEs) at the BS and fixed number of CUEs, DUEs, and channels is considered in [67]. A bipartite graph connects the sets of CUEs and channels, the edges’ weights being the average throughput achieved for each separate allocation. Afterwards, the CUEs and DUEs are connected via an undirected graph, with the received signal for each connection describing the respective weights. Due to the lack of CSI, however, these weights need to be estimated separately. Then, the most appropriate allocations are made by describing every possible link between each DUE and all CUEs in a bipartite graph and finding those with smallest weights. Accordingly, clusters are formed, with each one containing only a single D2D pair. Power allocation is performed for each cluster via a Nash equilibrium potential game, the solution of which is estimated via a Q-learning method. A relatively stable utility function $\Omega_{U}$ is obtained. A different approach to the RA problem is taken in [68]. Allocation of multiple RBs to a D2D pair is considered, with the limitation of preserving the CUEs’ desired throughput, while their channels are preliminarily allocated by the BS. The D2D pairs and available RBs are connected via a bipartite graph, the edges’ weights being expressed by the proportional fair (PF) metric, which is a function of the achievable throughput. Multiple RB allocation is described by the same graph with the modification of repeating the vertices corresponding to the DUEs; $T_{RB}$ times ($T_{RB}$ is the maximum number of RBs per D2D link). The scenario is comprised of a fixed number of CUEs and channels, and a variable number of DUEs within the coverage of a single macro BS. It is notable from the results that DUEs throughput declines with the distance between them, but increases with their number for $T_{RB}=1$. Increasing $T_{RB}$ yields slightly better fairness that remains stable. A multiple small-cell scenario with a fixed number of channels and variable number of CUEs and DUEs is used as a base for the proposed distributed RA in [69]. A small cell serves different CUEs and DUEs, with the algorithm aiming to provide the necessary resources and minimize the interference between the cells and among the nodes associated with each one. The network is described as a graph with the small cells being its vertices, two of which are connected with an edge, if their mutual interference is greater than a predefined threshold. Then, channel allocation is performed via the Hungarian algorithm, subjected to maximizing the CUE satisfaction parameter (dependent on the user’s throughput). Afterwards, channel selection for the small cells is performed via a potential game, while a coalition game is used for channels sharing between the CUEs and DUEs in each cell. Nearly exponential growth of $R_{T}$ with the increase in $N_{DUE}$ has been reported. The authors of [70] proposed a new graph-based clustering solution that achieves what they term “pure D2D”, which describes channel allocation for DUEs without the involvement of the CUEs, in order to avoid mutual interference between the users, especially at the cell’s edge. In addition, some RBs are only allocated to DUEs, multiple DUEs may utilize the same RB, and the RBs of the CUEs are orthogonal. The scenario includes a fixed number of CUEs and channels, and a variable number of DUEs. The network is modeled as a graph, with the CUEs and DUEs being the vertices, and edges existing between every two nodes that can potentially interfere with each other. Then, all nodes are grouped in clusters so that there are no shared RBs within a cluster. After the clusters’ formation, they are refined so that nodes with better channel conditions may be chosen within each, through appropriate power allocations to meet their SINR requirements. This cluster reformulation is modeled as a tree that includes all potential nodes which should either be added to a cluster or not, depending on whether the SINR constraint is satisfied. As seen from the simulation results, the algorithm achieves a linear increase in the number of supported links, with the number of DUEs, while both the average throughput per user ${\bar{R}}_{u}$, and the fairness β remain stable.

### 5.6. Lessons Learned and Trends in Development

Based on the review of graph-based solutions for RA in underlay D2D networks, presented in this section, the following characteristics of current and challenges for future research, can be summarized:

- Significant emphasis is placed on the modeling of the network as a graph whereas the optimization algorithms are similar in principle. *The main challenge, then, is to define a graph which corresponds well to the real-world relationships between the network nodes and the available frequency and power resources.* On the other hand, introducing new modes of communication (such as NOMA or FD [60,62,63]) or application scenarios (such as V2V and V2I [55,56,57]) can expand the graph’s representation potency. Most of the reviewed methods utilize interference or bipartite grpahs due to their emphasis, either on the mutual influence between CUEs and DUEs, or on the allocation relationships between the nodes and the channels. Usually the solutions are divided into sub-problems which are solved in a particular sequence.
- Social graph and hypergraph structures are notable in some works. The social graph [66] has the benefit of incorporating not just the physical characteristics of the network’s nodes, but the user-related ones as well. Therefore, *it is better suited for application-driven algorithms (such as cellular BS offloading), which also underlines its main design challenge, i.e., the definition of the user’s social relationship, so as to be described via attributes of the vertices/edges.* The disadvantage of the social graph lies in the determination of the relationships of interest between the users in a D2D network. *They are not directly related to the physical relationships between the communication nodes, and need to be modeled separately, thus potentially increasing the computational cost of the RA algorithm.* The hypergraph, although harder to define, more completely captures the interference and movement dynamics between the incumbent and underlay nodes in the network. *It also has the potential for matching not just multiple types of nodes, but also channel and power allocations at the same time. This model has been shown to be of particular usefulness in vehicular D2D scenarios* [55,56,57].
- *As the number of nodes within a macro cell increases, graph-based methods may cause a very significant delay in order to reach convergence* [73]. Hence, they may be less suitable for scenarios where UEs change their mode from cellular to D2D due to the decreased performance for QoS in the high layers of OSI. *Consequently, it is desirable for graph-based solutions to be evaluated through high-layer performance metrics as well.* Another common limitation of the graph-based solutions is that *each cellular user utilizes only one channel, which is usually not the case in realistic deployments. If different numbers of channels for each user are considered, the resulting graphs may be overly complex, both in their definition and solution.*
- Some open questions concerning graph-based methods in D2D networks pertain to (1) *The extent to which they are scalable, and at what D2D deployment density does a method become unreasonably complex.* The graph’s topology may provide hints (such as maximum weight, maximum degree, and evaluation of the number of vertices at which the method converges in a reasonable time) as to how to determine such critical density. (2) The delay and the bandwidth needed for overhead exchange in large D2D networks are not usually considered directly. *It would be reasonable to assume, nonetheless, that it may be prohibitive, even if the RA algorithm is itself computationally light*. (3) The lack of perfect CSI at the BS, which may be alleviated through ML. However, *a sufficient amount of training data will be necessary, the generation of which may not be trivial.*

## 6. Graph-Based RA in CR Networks

**Figure 6:**
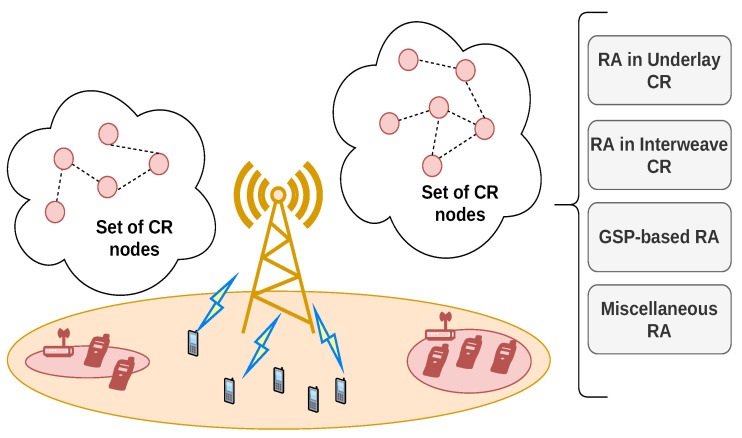
Scenario of CR networks and summary of methods for graph-based RA.

The CR devices take advantage of SDR-empowered transceivers that enable DSA for mutually beneficial SSh between themselves and the primary users (PUs) of the spectrum, i.e., those which are incumbent to it by a license. Protecting their communications while providing better spectrum utilization in time, frequency, and space, is the fundamental problem of CR networks (their nodes are referred to as secondary users, or SUs) [74]. The SSh between primary and CR networks is achieved either by an autonomous operation (interweave mode) of the latter from the former, or through cooperation (underlay, overlay, and hybrid modes) for their mutual benefit. SUs usually need to first determine which channels are available through spectrum sensing, and afterwards distribute these frequency resources among themselves. Depending on their mode of operation, they may be allowed to transmit on channels used by the PUs under an interference constraint. These design considerations have made the application of graph theory somewhat limited in current literature, but the available works reveal these algorithms’ potency for solving diverse SSh and RA problems in CR networks, especially in the underlay mode. The scenario of CR networks is illustrated in Figure 6. It also includes a generalized illustration of the most common graph model for RA problems, and a summary of their subcategories in this Section. A tabular summary of the main design characteristics of the algorithms reviewed in this Section, and the assumptions they are developed under are given in Table 8.

### 6.1. RA Methods in Underlay CR Networks

A graph-coloring based dynamic channel allocation solution for an underlay CR mesh network, constrained by the SINR of both the PUs and SUs, is proposed in [75]. Each PU is served by a separate BS, while a single CAP serves all the SUs, with the CAP coordinating with the BSs. The CR network is modeled as an undirected graph which connects each pair of SUs if they have at least one common channel in their sets of perceived available channels. RA is performed via graph coloring on a conflict graph for each SU, which has the set of adjacent nodes as well as the set of edges (of the original graph), connecting that SU to its neighbors as vertices. They are connected via edges, which represent the SINR of each link. RA is performed through graph coloring that minimizes conflicting channel allocations between the links. The total throughput $R_{T}$ (combining the throughputs of both PU and SU networks) increases linearly with the number of SUs. An alternative underlay CR scenario, with each SU being assigned only one channel, is considered in [76] for an edge-cutting RA method that aims to maximize the gains of both PUs and SUs. The channel allocation problem is modeled as a bipartite graph that connects the SU pairs and the channels, with the edges’ weights being the utility function depending on the SUs’ throughput and interference generated to the PUs. Then, each SU forms a preference list of desired channels, which is used to filter out channel allocations (edges) with low throughput potential. In this way, a smaller set of channels that have stable CSI is determined to maximize the SU’s throughput. The proposed solution retains nearly constant error due to CSI variations, with the increase in the maximum number of edges. Clustering-based RA in an underlay CR network, subject to dynamic constraint for the interference perceived by each PU, is explored in [77]. An interference graph describes the CR network connecting the SUs, with the edges’ weights being the channel gains. Then, the SUs are clustered such that their perceived channels have similar gains, and at the same time, the clusters’ sizes are optimized to prevent inter-cluster interference. This is achieved by cluster centers chosen among the SUs, based on their channel quality. After the clusters are formed, channel and power allocations are performed separately. The total CR throughput increases exponentially with the number of channels and the maximum transmission power of the SUs.

### 6.2. RA Methods in Interweave CR Networks

The authors of [89] consider the RA problem in interweave CR networks as a clustering of the SUs that have perceived common sets of channels as available for their communications. This formulation introduces conflicting objectives—clustering fewer SUs will yield higher throughput because they will have the similar sets of available channels, but at the same time, the number of clusters will be greater, which will increase the overhead. The cases of perfect (provided by spectrum availability database) and imperfect (via distance-dependent spectrum sensing) spectrum occupancy information are considered. Clustering is performed via biclique graphs (complete subgraphs within a bipartite graph) that are formed from bipartite graphs constructed by each SU, which connect the channels perceived by it, and all its neighboring SUs, as available. Then, three criteria for optimal matching of the SUs and channels are examined. For each SU, the number of matching channels is obtained and used as a characteristic, on the basis of which, via communication between the SUs, the clustering is performed. The number of bicliques (clusters) of certain size, the probability that one or more clusters are formed, and the average cluster size are the performance indicators for this algorithm. The cluster formation probability grows exponentially with the probability of channel availability, while it declines in the same manner as the biclique parameters increase. An interweave CR network with MIMO-enabled APs and single-antenna SUs is explored for the RA solution in [78]. Perfect channel occupancy knowledge at the CAPs is assumed, some SUs are not assigned a separate channel, and power control is not considered. The CR network is modeled as a graph with the SUs being vertices, with edges connecting two SUs if the degree of orthogonality between their channel gains is higher than the predefined threshold. To avoid interference with the PUs, each vertex is characterized by a weight coefficient which is the ratio of the norm of the channel gain vector between the SU and all PUs, and the average channel gain on each channel. RA is performed via a greedy graph coloring algorithm, which also considers the number of SUs that can be assigned to each channel. Only SUs, with a degree of orthogonality toward the PUs, with the PUs which are over a certain threshold report their feedback to reduce the overhead. The proposed solution’s convergence time increases linearly with the number of SUs, while the total CR network throughput $R_{CR,T}$ grows exponentially.

### 6.3. RA Methods Based GSP

A GSP procedure for DRL-based cooperative spectrum sensing (CSS) of the PU signal is developed in [79]. Each SU is a vertex in the coordination graph which establishes connections among all nodes, the decisions (on spectrum occupancy) of which influence the throughput of their neighbors. Then, the problem is defined as maximizing the CR network’s potential throughput, subjected to the SUs’ decisions. These are exchanged between the graph’s vertices via message passing so as to determine the optimal decision (action) to train the DRL algorithm. Fast convergence to very good probabilities of misdetection $P_{MD}$ and of false alarm $P_{FA}$ is observed. A DRL procedure for GNN generating joint channel and power allocation for the throughput maximization of an underlay CR network is developed in [80]. The graph is composed of the associated links (each user is constantly connected to the same AP) as vertices, with the interference links between them being their edges. The vertices’ attributes (which are the GNN’s inputs) are the distance between the PUs and the SUs, as well as the channels they occupy. With this data, the GNN produces channel and power allocations for both the SUs and PUs. Then, the DRL procedure ensures the PUs’ throughput requirements are met. The proposed algorithm achieves stable network throughput $R_{T}$ that is much higher than that of alternative learning procedures.

### 6.4. Miscellaneous Methods

The authors of [81] study channel and power allocation for a CR network which is comprised of stationary CAPs and SUs, with the goal of maximizing the number of SUs served $N_{SU}^{\prime }$ while imposing constraints on the minimum DL SINR for the SUs $\gamma_{SU,min}$ and maximum DL interference towards the PUs $I_{PU,max}$. Each SU can only occupy a single channel, the power level for which is chosen so as to provide $\gamma_{SU,min}$ according to the path loss (this is coordinated among the CAPs), and all CAPs have information about the SUs (all of them are active). The CR network is represented via a graph, the vertices of which are the SUs, and edges connecting the interfering vertices. It is constructed at the start and its channel allocations change to adjust to the requirements for $\gamma_{SU,min}$ and $I_{PU,max}$. Simulations show that $N_{SU}^{\prime }$ declines linearly with the increase in the number of PUs $N_{PU}$, of cognitive APs $N_{CAP}$ and channels $N_{c}$. This work is expanded in [82] by considering the availability of only local knowledge (each CAP only has information about the SUs within its range). First, the SUs with the smallest channel gains are assigned for each CAP to define the coverage matrix, and power allocation is performed for each CAP-SU link. Then, the active SUs and available channels form a bipartite graph, and the number of disjointed edges is maximized to perform channel allocation. Over 10% more SUs are served using this method in comparison to that in [81], while their number declines exponentially with the increase in PUs. A CR relay network (it includes nodes that serve as relays for the SU’s communications) with joint relay selection and channel allocation is considered in [83]. The proposed solution aims to simultaneously minimize the sum transmission power of the CR network and maximize $N_{SU}^{\prime }$. The channel allocation problem is described via a bipartite graph with the CR transmitter–receiver pairs and the channels as vertices, with the edge’s weights representing the sum of CR transmitter and CR relay output power levels among the set of all relay selections for a particular channel. The heuristic algorithm determines the selection of relay nodes and of channels that minimize this sum. Simulations show that the sum of the CR network’s total transmission power $P_{CR,T}$ does not grow over 20% higher than that of the PU network. Channel distribution with the probability of conflict reduction for a CR network is considered in [84]. A graph connects all SUs which have links between each other. The channel distribution is performed using a star graph that has the channels and links between the SUs as vertices, with the edges’ weights representing the conflict probability that depends on which channels are perceived as available by each SU. Three heuristics algorithms are introduced to solve this problem, with the node-link-based channel allocation (optimal matching between channels and adjacent links) yielding the highest percentage of assigned SU links, remaining relatively stable with the increase in $N_{PU}$. A similar scenario is considered in [85], considering both centralized and distributed RA, while protection of the PU communications is facilitated by the list of unavailable (blocked) channels at each SU pair. This list is the attribute of the conflict graph that connects all SU pairs, and their edges’ weights are comprised of both the co-channel and adjacent channel interference for each potential conflicting allocation between two links. These attributes combined together form the SU saturation metric, on the basis of which vertex coloring is performed so as to assign the channels. The solution’s convergence time increases linearly/exponentially with the number of SUs for the distributed/centralized variants. However, the average throughput per SU ${\bar{R}}_{SU}$ declines exponentially. A CR-based IoT network is considered as a scenario with the aim of optimal end-to-end routing for the SUs, in [86]. The SUs are modeled as vertices of a graph, with the links between them being the edges and the weights being the SINR per link. A genetic algorithm obtains the optimal channel allocations, with the power being fixed. The IoT network throughput is shown to decline exponentially with the increase in spectrum utilization (longer routes among the SUs). Optimizing EE and throughput in a CR sensor network comprised of CAPs and SU sensors is considered in [87]. The network is modeled via a hypergraph, the vertices of which are the SU sensors, and its hyperedges encompass ordered subsets of both the interfering and interfered SUs, their received powers constituting the edges’ weights. Then, the task is formulated as channel allocations, such that the utility function $\Omega_{U}$ (comprised of both the EE and throughput) is maximized. Fast convergence is achieved. RA in a CR network is studied by the authors of [88]. A graph describes the conflicts (of associations with the same CAP, and on the same channel) between the SUs, with the edges’ weights representing the potential mean throughput of a particular allocation and association. Two variants of the algorithm, with either assumed mean throughput per connection or its estimation, are developed. When they are compared in the simulations, the latter variant exhibits logarithmic decline in its performance, as the numbers of SUs, CAPs, and channels increase.

### 6.5. Lessons Learned and Trends in Development

On the basis of the review in this section, the following observations and research directions can be denoted:

- *The mutual influence between the PU network and that of the SUs (which, in a realistic scenario, may be significant) is rarely considered.* Usually, the CR network is described as a separate graph. The SUs’ available channels are assumed to be fixed, and consequently, only the SUs’ throughput is assessed. However, in this case, the interference originating both from the SUs to the PUs, and the other way around, as well as the spectrum utilization gain (The throughput increase related to the introduction of the CR network in the spectrum of the PU) is not accounted for. *The difficulty, then, comes from the graph and optimization algorithm designed to consider the RA of both networks, and (depending on the CR’s mode of operation) coordinate the SSh and RA decisions to provide optimal spectrum utilization and protection of the PU communications. A suitable tool for this purpose is for them to be modeled via GSP.*
- The assumption of perfect CSI/spectrum occupancy information is problematic, especially in CR networks, which are not licensed users of the spectrum. PU misdetections should be considered in graph-based RA for SUs. Their $P_{MD}$ and $P_{FA}$ (as functions of the measured SNR) may be used as valuable attributes in a graph. Respectively, mechanisms that reduce the influence of imperfect spectrum sensing are needed. As presented by [79], *high-accuracy CSS can be implemented as a GSP-based solution, and thus included in the procedure.*
- Depending on the CR operation mode and the SUs’ number, the overhead exchange for clustering and RA may be prohibitive. Moreover, the RA method should consider the variable number of channels for SUs, which are characteristic of interweave CR networks. *A disadvantage of graphs is that their number of vertices is fixed, and thus, their definition for modeling allocation of rapidly varying availability of channels (within the duration of the same simulation instance), may be significantly more difficult. Therefore, the graph-based solution may include UL/DL decoupling on different modes (DL on interweave, featuring non-graph based optimization, UL on underlay), with preliminary training of a GSP-based DRL method for specific deployment scenarios.*
- The UA and RA of SUs/CAPs should be designed with consideration of whether the CR nodes are stationary, airborne (on UAVs), ground-based vehicular, marine, or carried/worn by humans [74]. They are situated in different locations/heights and diverse radio propagation conditions, so the complexity/capability of their functionalities for spectrum sensing, clustering, UA, and RA should reflect the environment’s severity.

## 7. Challenges and Design Aspects of the Holistic Graph-Based Resource Allocation for Integrated Space Terrestrial Networks

A substantial number of recent works [1,2,3,90,91] have provided important visions for 6G ISTN architectures and their requirements in terms of applications, technologies, hardware, signal processing, and resource distribution. It is envisioned that 6G will not simply include different kinds (in terms of coverage, throughput requirements, and node density) of terrestrial (TNs) and non-terrestrial networks (NTNs), but will incorporate them into a global system. This will be driven by the mutual benefits of network convergence, for example, UAVs providing backhaul for densely deployed terrestrial APs, or low-orbit satellites broadcasting common data streams to groups of users that require the same content [92]. Consequently, a solid basis is established, for the development of algorithms for RA, multiple access, EE, and security, operating in the relevant application scenarios. They are diverse, incorporating marine, terrestrial, air, and space communications, as well as many types of devices (some of which support multiple RATs) with various capabilities and throughput/latency requirements. This section aims to facilitate the RA for these communication networks by considering (1) the existing 6G ISTN concepts; (2) graph-based algorithms for RA for different types of networks, and (3) their mutual coexistence on a large scale on the ground, in the air, underwater, and in space. Challenges for algorithm design within the proposed resource management framework are also described.

**Figure 7:**
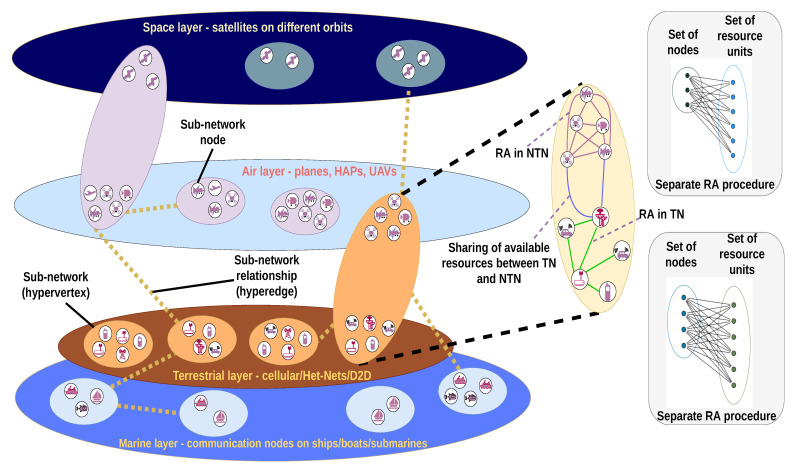
The GRIST concept.

Architectures for 6G global inter-connectivity and network coexistence have considered multiple kinds of connected devices that are categorized by the environment they operate in. Sixth-generation integrated communications consist of a multi-layered structure containing marine, terrestrial, air, and space environments (some of them can contain several tiers; for example, different satellites operate on predetermined heights [92]), as illustrated in Figure 7.

This demonstrates the proposed concept for GRIST, depicted as a set of hypergraphs composed of different sub-networks on every layer, and the connections between those of them that interact with each other for the provision of their required services. A sub-network may be formed from communications nodes of the same type, i.e., terrestrial/aerial/ space, or a combination of these, to describe the integration of TNs and NTNs conceptualized in [93]. The sub-networks are described by separate graphs (for simplicity, only their vertices are shown, as white circles containing example communication nodes), and may be formed around a coordination point (CP) that determines the sub-network’s range of operation. This CP is a network infrastructure unit that incorporates the communication flows within the sub-network so as to facilitate a specific application scenario. Hence, it may be realized as an AP that provides access to all wireless nodes within a home, or as an RRH/UAV-based AP/cellular macro BS/satellite-based node that covers a particular area with its UEs, BSs, ground-based APs, and others (such as V2V nodes, IoT devices, or patches of large intelligent surfaces). In coexisting incumbent and cognitive sub-networks, the CPs may be implemented in the Spectrum Access Control units that are responsible for RA and network self-organization, as described in [74]. As illustrated in the GRIST concept (Figure 7), the sub-networks (the ellipses within each layer containing the communication nodes) are represented by the hypergraph’s hypervertices. While individual sub-networks may resolve their UA and RA autonomously, most of them will collaborate/share spectra with each other, which is described via the hyperedges between them. The management of resources and their allocation is determined within the span of a single hypergraph that connects sub-networks covering a particular volume of space during the time of a communication exchange which requires the sub-networks’ mutual collaboration (Other communications tasks, such as user data processing, channel estimation, and signal and traffic flow classification, may be more efficient if they are realized via distributed learning methods [94]). A conceptual example of this cooperation within GRIST is given on the right side of Figure 7, which illustrates an enlarged version (in light yellow) of one hypervertex. It shows the modeling of the sub-network cooperation by the vertices (nodes of a TN and a NTN) and the edges that connect them. These connections are depicted in green (model of the RA in TN), violet (model of the RA in NTN), and blue (sharing of the available resources of the TN and the NTN, performed by the CPs of the two sub-networks). The edges within the TN and the NTN are determined by the physical and social relationships between the nodes (such as channel/power allocation, SSh, EE). Additional RA problems that are solved separately (often being the distribution of available channels) for each sub-network may be performed via a bipartite graph, as is common in the literature. They are defined for the sub-network’s set of nodes and the set of respective resource units. The hypergraph illustration of the relationships between the sub-networks in Figure 7 gives the following information that can be utilized in formulating and solving the RA problem:

- Hypervertex attributes:
  - Available/unutilized channels. They may be shared with sub-networks of the same type or via CRs. In addition, CPs in nearby sub-networks can use this information to regulate their power and channel allocation.
  - Resource demands to meet the sub-network’s QoS requirements. A supplementary attribute that provides greater agility in SSh pairing between sub-networks. It also establishes priority in resource distribution due to the requirements (for example, groups of users requesting the same high-throughput streaming content, holographic telepresence, etc.) of certain sub-networks.
  - Sub-network EE gain. Its purpose is to describe the sub-network’s overall performance to notify its neighbors of the need for channel/power allocation readjustment. Furthermore, some operator infrastructure nodes (BSs/APs) may be switched to idle mode so as to reduce the power consumption. Achieving high EE will be the main optimization goal of the GRIST.
  - User data characteristics. Cooperative caching between sub-networks (both terrestrial and non-terrestrial) is facilitated by information about which data (*type*, *amount*, and *period of time* to be cached at a particular node) are required in a sub-network (or a set of sub-networks). This information is acquired through ML.
- Hyperedge attributes:
  - Distance between sub-networks. Determines the viability of frequency reuse and communication quality due to path loss. Unused channels can also be shared within a distance such that the potential interference is avoided. In addition, path loss is a significant consideration in communications between terrestrial and air/space nodes, so their association is dependent on the distance.
  - Carrier frequency of the connected sub-networks. It is an additional factor, together with the path loss, for association, resource sharing and information exchange between two sub-networks, especially considering the frequency range expansion with mmWave and terahertz in beyond 5G.

Through these features, the resulting optimization problems for a set of sub-networks within a hypergraph consider the results of their individual RA procedures for the overall resource management. Thus, they present a strong incentive for developing global solutions through GNNs based on message passing and vertex aggregation [11,18,95]. Hereby, the limitations of the proposed GRIST concept are identified as follows:

- The implementation of the RA procedures in ISTNs naturally involves a large number of nodes, sub-networks, and the optimization of the relevant parameters. Therefore, defining and solving graph-based RA algorithms in such scenarios is limited by the number of communication nodes that will ensure feasible computational complexity for the devices’ processing capabilities. This also poses the question of the optimization method’s scalability for realistic scenarios.
- Consequently, the implementation of RA will be limited by the requirements for EE of the sub-networks’ nodes.
- The RA algorithm for GRIST is unlikely to employ a unified graph model to describe the sub-problems that need to be solved due to the variety of the design parameters and radio environments in the different layers. As a consequence, the design process will be overburdened.

**Figure 8:**
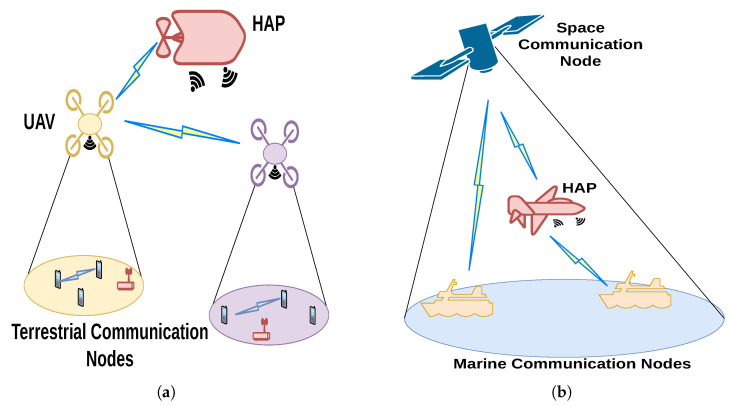
Illustrations for sub-networks for communication exchange over terrestrial, marine, aerial and space layers. (**a**) Sub-network comprised of terrestrial and aerial nodes. (**b**) Sub-network comprised of marine, aerial, and space nodes.

Following these limitations, several open challenges and research directions for the design and implementation of graph-based RA procedures are expanded in a greater detail:

- **Modeling coexistence and communications between sub-networks on different layers of GRIST.** As it has been explored in various studies [90,91,93,96,97], the realization of the coexistence and reliable information exchange between contemporary and future networks is non-trivial. In order to provide ubiquitous and saleable networks that ensure seamless communications on all four layers [93,96], the integration of TN and NTNs can be facilitated by graph-based algorithms for RA, energy conservation, data offloading, localization, and dynamic spectrum access in the unlicensed bands (that are suitable for upcoming aerial communication nodes [97]). Such solutions will need to consider the physical parameters (speed, direction, weight, altitude of operation, size, available power supply/battery, etc.) of the communication node, which, together with the environmental aspects (such as fading, shadowing, wind, rain, and distance-dependent path loss) affect the communications. These aspects will then define the graphs’ structures and attributes within GRIST. The following two examples are given: (1) RA for a sub-network that includes terrestrial and aerial nodes (Figure 8a), is dependent on the physical parameters of the UAVs mentioned above, as well as on the movement speed and the spectrum access scheme (fixed/dynamic) of the ground users [96,97]. Together with the traditional RA problems in a terrestrial network, the graph-based solutions within GRIST will need to solve the problems of user and UAV mobility, determined by the aerial nodes’ battery limitation, transmission power, and available channels (incumbent or shared), which can also be described as vertex attributes. (2) The most essential parameters that concern the RA for a sub-network comprised of terrestrial/marine/aerial and space nodes (Figure 8b) are the link delay, achievable data rate, and the duration of direct visibility between the nodes [98]. They can be used as edge attributes, while physical parameters (such as longitude, latitude, speed, and capacity) serve as vertex attributes for graph-based RA algorithms for ISTNs.
  The optimization problem can be solved using deep neural networks and reinforcement learning (RL)-based training procedures, which have shown promising performance gains (such as fast convergence and short processing time) [99]. Different learning procedures may be utilized depending on the operations for service provision in the sub-networks. Supervised learning has widely been used for functions such as signal decoding, spectrum sensing, and multiple access, while reinforcement learning has been applied for RA on a sub-network level, and for radio access technology selection for ISTN [100,101].
- **Modeling of graphs for RA methods in GRIST sub-networks.** The relevant types of graphs, including GSP (Section 3), which is used for the modeling of the reviewed RA methods in each sub-category (comprising Section 4, Section 5 and Section 6), are highlighted in Table 9 that matches them with the sub-categories. *The table illustrates which of them are most commonly applied, as well as those appropriate for application in GRIST.* Firstly, as noted in Table 9, applying a graph-based RA approach will likely model both the sub-network’s nodes as a general/complete graph, and connect the available channel and/or power resources and users as a bipartite/tripartite graph. The general/complete graph type is the most intuitive for modeling the physical relationships between the network’s nodes, as their parameters (power level, channel gain, distance, interference, etc.) are aptly considered. In this way, the optimization method will also be updated at each iteration with inputs that reflect the influence of the nodes’ movement and the changes in their radio environment. The main limitation of this type is the significant increase in complexity with the number of nodes. Bipartite/tripartite graphs allow for resource distribution that also considers the environment’s influence, and they are easier to define. Nevertheless, they are viable for a fixed set of resources that are only available to a particular sub-network. Thus, these two types of graphs are near-universally applied in RA methods, as exemplified by the review in the previous Sections. Depending on the optimization method’s complexity (contributed primarily by the nodes’ density, the number of their links (i.e., whether they implement MC), the number of frequency channels, the social relationships between the nodes, etc.), an edge reduction mechanism will likely be necessary to increase the algorithm’s convergence speed. Overhead exchange for resource availability between sub-networks, as well as the management of resources for multiple sub-networks, may be modeled via star graphs, as they can describe the consolidation of the overhead in a single node (such as the CP). The current advancements in deep learning and its prominence in wireless communications research [12] point to the potential for RA algorithms within GRIST to be trained using GSP/GNN. In general, path graphs are not appropriate for modeling of RA methods for GRIST due to their linear, non-hierarchical nature.
- **Solving algorithm.** As seen in the review in the previous sections, heuristic algorithms based on logical sequence of procedures are prevalent in graph-based solutions for wireless communications. In addition, complex RA algorithms are often decomposed into sub-graphs or a combination of graph-based and traditional optimization methods. It is questionable to what extend such approaches will be practical, in terms of efficiency and performance, for implementing GRIST on a wide-scale. Therefore, scalability as the number of connected vertices grows substantially, even within a small area in a single layer of the ISTN, is a primary research direction for the development of GNN-based learning methods for RA between sub-networks. Although not a universal solution, graph encoding and compression [102] may be appropriate alternatives for application in GRIST. Furthermore, a recent method for sequential graph construction and aggregation [103] for GNNs has shown significant reduction in memory usage and processing time.
- **GNN-based solutions for sub-network functionalities.** Deep learning on graphs has shown potential for not just RA, but also for cognitive functionalities such as cooperative spectrum sensing, spectrum database construction, localization, modulation recognition, and spectrum decision [79,104,105], due to its ability for feature (SNR, distance, achievable throughput, QoS requirements) extraction from the network’s nodes. The impact of deep learning on graphs for these functionalities depends on the sub-network’s application, density, and throughput requirements. For the implementation of some of them, it may be more feasible to use a combination of probabilistic and deep learning methods, or the employment of the same type of data (such as received signal samples or statistics of various performance indicators) to train multi-purpose neural networks. Accordingly, multiple tasks (such as transmitter detection and recognition) may be performed both by the CP, or distributively (by sensors/APs/UEs) via the same deep learning model, while the achieved results can yield the information parameters (spectrum occupancy, user density, etc.) that form the hypergraph attributes described above. Then, the RA problem may be solved through reinforcement/supervised learning of a neural network on a graph.
- **Applying FL for RA between sub-networks.** The novel FL concept allows for decentralized learning of a global model by multiple devices in the network that perform training on their local datasets and transmit only the resulting parameters to the aggregation unit (usually a cloud server) [106]. In this way, the overhead exchange is reduced and the protection of the users’ private data is increased. Thus, the CSI and other traffic information can be provided from the sub-network’s UEs to their serving CPs, which perform continuous learning of their local models. Cooperating CPs can collaborate by aggregating just their individual RA decisions/results or GNN parameters (such as in [50]) at higher-level nodes within a network’s architecture (such as a remote cloud server of an IoT/cellular network).
- **A significant challenge in the implementation of GRIST is the overhead exchange between the CPs and among the sub-networks’ nodes.** It can be reduced through decentralized prediction of the wireless nodes’ movement behavior, traffic, and link delay [92]. This is made possible by the availability of information at each node, as well as the requirements and conditions of the vertices connected to it [95]. The prediction process can be further incorporated into a FL scheme for sub-network RA. It has been envisioned that the power consumption of the incurred overhead exchange will be reduced through renewable energy sources, dedicated to the wireless nodes [107]. Furthermore, compression of the redundant overhead information has also been recognized as an important research direction for decentralized learning in 6G.

**Figure 9:**
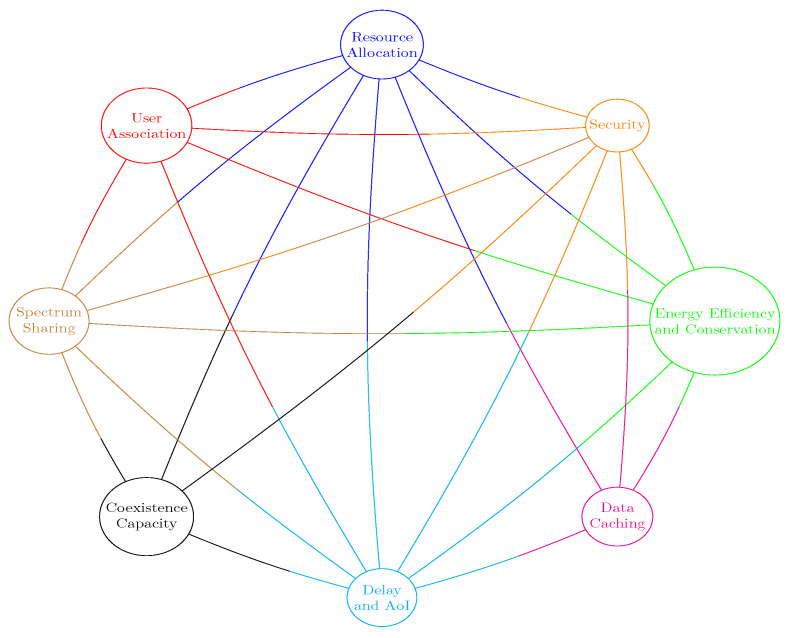
Graph-based model of the design process for resource management algorithms (adapted with permission from Ref. [108]. 2020 IEEE).

In addressing these challenges, the design process of RA algorithms can be viewed as an optimization problem by itself, which can also be modeled via a graph and driven by particular network application (IoT/cellular/air and space communications, and so on). The graph-based model of the design process of ultra-dense networks (UDNs) from [108] is here further developed (Figure 9) to incorporate the dependencies on application requirements for RAT, BS/AP/UE densities, throughput, and delay in ISTNs. These factors, as well as the respective scenario a network operates in, will determine the weights of the edges that connect the different design aspects (vertices), with the vertex attribute describing the loss (cost) coefficients of neglecting a particular aspect (For example, the SSh vertex attribute describes the largest tolerable interference between PUs and SUs on particular channels, while the RA vertex attribute illustrates the required throughput to be achieved on the per user or network-wide basis, to ensure the efficient spectrum usage). To develop the concept presented in [108], this model expands the resource management and interference avoidance challenges that are more prevalent in traditional cellular and UDNs by including the RA, UA, SSh, and CC aspects, thus making its application broader. These four aspects comprehend the allocation, utilization, distribution of the available frequency, and power for inter-operability between various wireless technologies. They are also addressed by various methods such as network self-reconfiguration, resource distribution, multiple access, spectrum mobility, channel estimation, and spectrum sensing. The Delay and AoI, EE, and Data Caching are also addressed, in a broader sense, by the functions that ensure the efficiency of wireless networks in terrestrial, aerial, marine, and space applications. These functions require not only low algorithm complexity, but also agile energy usage depending on the traffic, definition of constraints in terms of time, and energy conservation through caching of specific data to APs that are close to the users. By finding the path with the best utility-to-cost ratio on the graph in Figure 9, the most important design considerations (so as to decrease the algorithm’s complexity) can be determined.

The graph on Figure 9 connects all design aspects that are directly dependent on each other, and the design process will require a suitable trade-off between them, so as to adapt the algorithm’s complexity and applicability according to a particular scenario. This model is a complete graph, although some relationships between the aspects and functionalities are too broadly related to each other, and the edges they constitute can be neglected. The edges coming out of each vertex are colored in a combination of the connected vertices’ colors (denoting the vertex attribute), so as to illustrate the weight of each edge. The design graph’s vertex concerning the RA (the leading problem that concerns spectral efficiency, and thus related to all the rest) have been sufficiently clarified, so only the remaining aspects are hereby described.

- Spectrum sharing. Generally, it is considered a requirement for: (1) achieving the efficient utilization of the allocated spectrum of a particular communication system by sharing it with other wireless standards (it has been established by multiple measurement campaigns, such as [109,110], that the spectrum of many modern communication systems, especially those operating in the sub-6 GHz range, is poorly utilized for most of the time. Therefore, the coexistence between different wireless technologies is presently considered as a major research topic); and (2) integration of communication systems operating in different (especially, adjacent) frequency bands [111]. In other words, SSh is performed: (1) between the incumbent (primary) and non-licensed (or secondary) users; and (2) between the primary users of different wireless technologies. This aspect is applicable mostly for CR networks, and related to all other aspects apart from Data Caching, because that is directly associated with EE and RA.
- User association. The upcoming and future networks are multi-tiered, i.e., users communicate via multiple types of cells or access points (APs), and they do not associate with them in the same way. In some applications, the users are attached to multiple macro cells, while for others, numerous APs serve a single user (especially in scenarios where they are much more densely deployed than the UEs). Thus, UA is generally relevant for EE, SSh, Security, RA, and Delay.
- EE and conservation. This aspect represents a design requirement for not only the hardware components of the BSs, APs, and the user terminals, but also the network self-organization schemes which they operate on. These algorithms need to be designed with the consideration of minimizing the power consumption, which will otherwise become intolerably large as the networks in 5G and beyond encompass an ever-increasing number of nodes [112]. EE balances the computational complexity of the RA and power consumption, and it is relevant to all other aspects except Coexistence Capacity.
- Delay and Age of Information (AoI). Traditionally, wireless networks have had different latency and throughput requirements depending on whether the transferred information is to be perceived by the recipient in real time or not (and also how “real time” is defined for a particular scenario). With the increasing connectivity between machines and cyber-physical systems, the delay requirements have become more diverse. Recently, the AoI as a more general metric has been proposed [113], denoting the cumulative timing delays that consider both the physical and communication mechanisms that comprise the operation of the network’s nodes. The AoI is dependent on the number of nodes and the maximum number of iterations for the communication process’ completion. This aspect is related to all the rest.
- Coexistence capacity. This aspect describes the extent to which the application permits/enables coexistence of the network with others of different RATs, and is associated with the nodes’ communication capabilities and the operator’s policies. It is the most important determining factor for the complexity of the resource management scheme. This aspect is related to RA, SSh, Delay, and Security.
- Data caching. If the deployment scenario (such as V2V and cellular offloading) requires caching at the UEs/CPs, its realization (by determining which nodes are to cache what information, and how they are clustered) may have greater importance than the EE. That is due to the power conservation gained from avoiding the unnecessary transmissions from the BS/AP to each user. This aspect is related to the RA, Delay, EE, and Security.
- Security. As the services provided by the wireless networks, as well as the RATs that facilitate them, become more complex and demanding, security protocols need to be additionally enhanced to provide user data protection and prevention of unauthorized spectrum access. Achieving this will require supplementary procedures implemented in the various nodes via FL-based distributed methods [107,114], and a non-negligible overhead that is used for encryption of user data, authorization, and jamming of the malicious users for protection on the physical layer and of the authorized users’ access to the spectrum. Consequently, it is relevant to all the other aspects.

## 8. Future Research Directions

In summary, the following directions for future research can be identified:

- The main challenge is decreasing the computational complexity of the RA methods by defining the graph in such a way as to decrease the number of vertices and avoid unnecessary connections between them.
- Using the proposed graph-based algorithm design model, the most prominent challenges for a specific network scenario and user terminal capabilities can be identified and focused upon.
- Incorporation of different functionalities that enable satisfactory communications and low power consumption.
- Integration of novel communication networks and services alongside existing ones through cognitive capabilities.

## 9. Conclusions

This paper introduces the GRIST model based on the current advancements of graph-based methods for RA applied in the three most prominent wireless network scenarios. Emphasis is placed on the development of their specific design features in terms of graph formulation and problem solving. GRIST facilitates the coexistence and inter-connectivity between various communication systems within ISTN, aiming to achieve network-wise maximization of the throughput and spectrum utilization. Its implementation considerations are described, together with a design process for such algorithms, based on a simplified variant of a complete graph, which determines the optimal balance between the underlying challenges.

## Figures and Tables

**Table 1:** Table of acronyms.

Acronym	Definition
5G	Fifth-generation wireless communications
6G	Sixth-generation wireless communications
ABRB	Almost blank resource block
AoI	Age of information
AP	Access point
BP	Belief propagation
BS	Base station
CAP	Cognitive AP
CP	Centralization point
CR	Cognitive radio
CSI	Channel state information
CSS	Cooperative spectrum sensing
CUE	Cellular user equipment
D2D	Device to device
DCA	Difference of two convex functions approximation
DL	Downlink
DRL	Deep reinforcement learning
DSA	Dynamic spectrum access
DUE	D2D pair
EE	Energy efficiency
FD	Full-duplex
GDFT	Graph Discrete Fourier transform
GNN	Graph neural network
GRIST	Graph-based Resource management for Integrated Space and Terrestrial communications
GSP	Graph signal processing
Het-Net	Heterogeneous network
ICI	Inter-cell interference
IoT	Internet of Things
ISTN	Integrated space and terrestrial network
KKT	Karun–Kush–Tucker
LTE	Long-Term evolution
MC	Multiple connectivity
MCNF	Minimum cost network flow
MIMO	Multiple-input–multiple-output
ML	Machine learning
mmWave	Millimeter wave
NOMA	Non-orthogonal multiple access
NTN	Non-terrestrial network
OFDMA	Orthogonal frequency division multiple access
OSI	Open systems interconnection
PF	Proportional fair

**Table 2:** Table of acronyms.

Acronym	Definition
PCE	Power consumption and externality
PU	Primary user
QoS	Quality of service
RA	Resource allocation
RAT	Radio access technology
RB	Resource block
SGD	Stochastic gradient descent
SINR	Signal-to-noise-plus-interference ratio
SSh	Spectrum sharing
SU	Secondary user
TN	Terrestrial network
TSR	Throughput satisfaction rate
UA	User association
UAV	Unmanned aerial vehicle
UC	User centric
UDN	Ultra-dense network
UE	User equipment
UL	Uplink
V2I	Vehicle-to-infrastructure
V2V	Vehicle-to-vehicle
WLAN	Wireless local area network
WSR	Weighted sum-rate

**Table 3:** Table of notations.

Notation	Definition
AR	Averaged risk-averse rate
A	Adjacency matrix
$\alpha_{u}$	Ratio of guaranteed users
B	Set of edges
B	Incidence matrix
β	Fairness index
*C*	Number of output channels
$\gamma_{T,CUE}$	Target SINR of the CUEs
$\gamma_{T,DUE}$	Target SINR of the DUEs
$\gamma_{SU,min}$	Minimum DL SINR for the SUs
ε	Energy efficiency
G	Graph
*h*	Scaling coefficient (weight)
$I_{PU,max}$	Maximum DL interference towards the PUs
$\theta_{u}$	Throughput satisfaction rate
J	Degree of connectivity
L	Laplacian matrix
$L_{u}$	Number of users per RB
Σ	Laplacian eigenvalues matrix
$\lambda_{AUE}$	Deployment density of AUEs
$\lambda_{MUE}$	Deployment density of MUEs
$\lambda_{RRH}$	Deployment density of RRHs
$\lambda_{UE}$	Deployment density of UEs
*M*	Number of edges
*N*	Number of vertices
$N_{UE}$	Number of UEs
$N_{BS}$	Number of BSs
$N_{DUE}$	Number of DUEs
$N_{CUE}$	Number of CUEs
$N_{SU}^{{}^{\prime }}$	Number of SUs that obtain service

**Table 4:** Table of notations.

Notation	Definition
$N_{PU}$	Number of PUs
$N_{CAP}$	Number of CAPs
$N_{c}$	Number of channels
$N_{AP}$	Number of APs
$N_{RRH}$	Number of RRHs
$N_{AUE}$	Number of AUEs
$N_{RB}$	Number of RBs
$P^{out}$	Outage probability
$P_{FA}$	Probability of false alarm
$P_{MD}$	Probability of misdetections
$P_{T}$	Total network transmit power
$P_{CR,T}$	Total CR network transmit power
*r*	Distance
$R_{CR,T}$	Total CR network throughput
${\bar{R}}_{u}$	Average throughput per user
${\bar{R}}_{c}$	Average throughput per cell
${\bar{R}}_{SU}$	Average throughput per SU
${\bar{R}}_{AUE}$	Average throughput per AUE
${\bar{R}}_{MUE}$	Average throughput per MUE
${\bar{R}}_{AP}$	Average throughput per AP
$R_{T}$	Total network throughput
${\bar{R}}_{T}$	Average network throughput
$T_{RB}$	Maximum number of RBs per D2D link
V	Set of vertices
W	Weight matrix
$\Omega_{U}$	Utility function
$\Omega_{s}$	Social utility function

**Table 6:** Tabular summary for graph-based RA in cellular homogeneous and Het-Nets.

Reference	Application	Graph Model Type	Tasks Solved via Graphs	Graph Formulation	Optimization Method	Performance Assessment
[19]	Channel and power allocation in OFDMA	MCNF	Channel and power allocation	All channels connected to each user	Simplex network	${\bar{R}}_{u}$ of 500 kb/s, and $R_{T}$ of 50 Mb/s at (4×2) MIMO and 120 UEs of 1 cell
[20]	Channel allocation and sharing of TVWS with LTE networks	Interference graph	Channel allocation	Vertices—BSs; edges—interfering BSs	Heuristic algorithm	$R_{T}$ of up to 1.2 b/s/Hz for 3 BSs
[21]	RA for OFDMA small cell network	Conflict/cordial graph	RB allocation	Vertices—UEs; edges—interfering UEs	Heuristic algorithm	Convergence for β, $\alpha_{u}$, and $\theta_{u}$ for 150 small cells and 60 RBs
[22]	Channel and power allocation in OFDMA HetNet	Interference graph	Channel and power allocation	Vertices—UEs; edges—interfering UEs	Heuristic method	${\bar{R}}_{u}$ of 6 b/s/Hz, and ${\bar{R}}_{c}$ of 2.5 b/s/Hz/W for 4 UEs per AP
[23]	Channel and power allocation in OFDMA HetNet	Interference graph	Channel and power allocation	Vertices—UEs; edges—interfering UEs	Heuristic method	${\bar{R}}_{u}$ of 2.5 b/s/Hz, ${\bar{R}}_{c}$ of 3.5 b/s/Hz/W, and $R_{T}$ of 350 Mb/s for 4 UEs per AP
[24]	Channel allocation in a femto-cell network	Conflict graph	Channel allocation	Vertices—BSs; edges—conflicting BSs	Heuristic method	$R_{T}$ of 600 b/s/Hz for 100 BSs and 2 channels per BS
[25]	RB allocation in an OFDMA Het-Net	Interference/ bipartite graph	Channel allocation	Vertices—UEs/user clusters; edges—between all UEs/RBs	Hungarian algorithm	$R_{T}$ of 12 b/s/Hz, and ${\bar{R}}_{u}$ of 0.1 b/s/Hz for up to 10 small cells, 100 UEs and 64 channels
[26]	RA for NOMA network	Directed graph	Power allocation	Vertices—UEs; edges—all UEs	Fast greedy algorithm	$P_{T}$ of up to 65 dBm for 300 users and 50 groups
[27]	RA for NOMA network	Bipartite graph	RB and power allocation	Vertices—UEs, RBs; edges—allocations	Heuristic algorithm, analytical solution	ε up to 46 kb/s/J for 24 users and 8 RBs
[28]	Channel and power allocation in a Het-Net	Factor graph	Power and channel allocation	Vertices—BSs and UEs; edges—between BSs and UEs with adequate SINR	BP	$R_{T}$ of up to 11 Mb/s, and ${\bar{R}}_{c}$ of up to 6 Mb/s for 20 UEs and up to 6 small cells and 1 macro-cell
[29]	Channel and power allocation in a mmWave Het-Net	Coordination graph	Power and channel allocation	Vertices—BSs and UEs/between BSs; edges—between BSs and UEs with adequate SINR/between interfering BSs	BP/RL	$R_{T}$ of up to 11 Mb/s, and ${\bar{R}}_{u}$ of up to 1.5 b/s/Hz for 8 small cells and 1 macro-cell
[30]	Channel allocation in a wireless network	Directed graph	Channel allocation	Vertices—BS and UEs; edges—between BSs and UEs within a certain range	Adam optimizer	$R_{T}$ of up to 8 b/s/Hz for 50 UEs
[31]	Power allocation in a Het-Net	Conflict graph	Power allocation	Vertices—BSs and UEs; edges—between BSs and UEs, and interfering BSs	RMSProp	Over 90% spectrum utilization for 8 BSs and 16 UEs
[32]	Power allocation in an ad-hoc network	Directed graph	Power allocation	Vertices—links between nodes; edges—between interfering links	SGD	$R_{T}$ of up to 90 b/s/Hz for 30 nodes
[33]	Power allocation in an ad-hoc network	Interference graph	Power allocation	Vertices—transmitters and receivers; edges—between associated and interfering links	Asynchronous SGD	${\bar{R}}_{u}$ of up to 6.5 b/s/Hz for up to 300 pairs
[34]	Channel allocation in a WLAN	Conflict graph	Channel allocation	Vertices—APs; edges—between APs within a certain range	RL/greedy algorithm	Over 90% spectrum utilization for 10 APs and 3 channels
[35]	RA and EE in HetNet	Interference graph	Channel and power allocation	Vertices—UEs; edges—interfering UEs	Gibbs sampler	${\bar{R}}_{u}$ of 1.8 b/s/Hz, and ε of 3 b/s/Hz/W for 96 UEs, with a dedicated channel for each
[36]	Channel and power allocation in a Het-Net	Interference graph	Power and channel allocation	Vertices—BSs; edges—interfering BSs	KKT	${\bar{R}}_{c}$ of up to 100 Mb/s (UL) and 80 Mb/s (DL), and ${\bar{R}}_{u}$ of up to 550 kb/s (UL) and 250 kb/s (DL) for 100 UEs and up to 4 small cells and 1 macro-cell
[37]	Channel and power allocation in an OFDMA Het-Net	Bipartite graph	Channel allocation	Vertices—BSs and UEs; edges—between BSs and UEs with adequate SINR	Hungarian algorithm, DCA	$R_{T}$ of up to 55 b/s/Hz for up to 350 UEs and 6 small cells and 1 macro-cell
[38]	RA for OFDMA Het-Net	Conflict/ cordial/tree graph	RB allocation	Vertices—cells/UEs; edges—interfering cells/UEs	Heuristic algorithm	Convergence for β, $\alpha_{u}$, and $\theta_{u}$ for 50 small cells and 50 RBs
[39]	Predictive RA for Het-Net	Conflict graph	Channel allocation	Vertices—BSs and UEs; edges—interfering cells and served UEs	Heuristic algorithm	$\theta_{u}$ of up to 80% and $R_{T}$ of 16 b/s/Hz for 30 UEs
[40]	EE, channel and power allocation	Bipartite graph	Channel allocation	Vertices—UEs; edges—channels	Dual Lagrangian algorithm	ε of 12 b/s/Hz/J for up to 5 UEs and 5 BSs

**Table 8:** Tabular summary for graph-based RA in CR networks.

Reference	Application/Assumptions	Graph Model Type	Tasks Solved via Graphs	Graph Formulation	Optimization Method	Performance Assessment
[75]	RA in an underlay CR network/CAP-BS coordination (realistic)	Undirected/ conflict graph	Channel allocation	Vertices—SUs/sets of links; edges—between SUs/between links; weights—SINR	Heuristic algorithm	$R_{T}$ up to 5 kb/s for $N_{SU}=45$, $N_{PU}=20$ and $N_{c}=16$
[76]	RA in an underlay CR network/Channel selection functionality (realistic)	Bipartite graph	Channel allocation	Vertices—SUs and channels; edges—between SUs and channels; weights—utility function	Heuristic algorithm	Channel allocation error close to 0% for $N_{SU}=N_{PU}=N_{c}=200$
[77]	RA in an underlay CR network/ Clustering-based organization (realistic)	Interference graph	Clustering of SUs	Vertices—SUs; edges—between SUs; weights—channel gain	Heuristic algorithm	$R_{CR,T}$ up to 3.5 Mb/s for $N_{SU}=20$, $N_{PU}=1$ and $N_{c}=120$
[78]	RA in an interweave CR network/Perfect CSI knowledge (unrealistic)	Undirected graph	Channel allocation	Vertices—SUs; edges—between SUs with sufficient orthogonality	Greedy algorithm	$R_{CR,T}$ up to 22 b/s/Hz for $N_{SU}=20$, $N_{PU}=3$ and $N_{c}=6$
[79]	CSS for CR network/Low $N_{SU}$ (realistic)	Coordinated graph	CSS	Vertices—SUs; message passing between SUs	SGD	$P_{FA}=0.06$ and $P_{MD}=0.04$ for a CSS among 4 SUs
[80]	RA in an underlay CR network/CAP handover not available (unrealistic)	Interference graph	Power and channel allocation	Vertices—associated links of the UEs and BSs; edges—between interfering links	SGD	$R_{T}$ of up to 5 for $N_{SU}=4$, $N_{CAP}=2$ and $N_{c}=8$
[81]	RA in a CR network/Stationary CAPs (realistic)	Interference graph	Channel allocation	Vertices—CAPs and SUs; edges—between interfering CAPs and SUs	Greedy algorithm	$N_{SU}^{\prime }=30$ for $N_{PU}=40$, $N_{CAP}=9$ and $N_{c}=8$
[82]	RA in a CR network/Local CSI knowledge (realistic)	Bipartite graph	Channel allocation	Vertices—SUs and channels; edges—between SUs and channels	Heuristic algorithm	$N_{SU}^{\prime }=30$ for $N_{PU}=25$, $N_{CAP}=4$ and $N_{c}=8$
[83]	RA in a relay CR network/User location knowledge (unrealistic)	Bipartite graph	Channel allocation and relay selection	Vertices—CR pairs and channels; edges—between CR pairs and channels; weights—transmission power	Heuristic algorithm	$P_{CR,T}$ is up to 20% higher than $P_{CR,T}$
[84]	RA in a CR network/control channel availability (realistic)	Undirected/ star graph	Channel allocation	Vertices—SUs/channels and links; edges—between SUs/between links and channels; weights—conflict probability	Hungarian algorithm	Up to 70% of links are assigned for $N_{SU}=15$, $N_{PU}=130$ and $N_{c}=30$
[85]	RA in an interweave CR network/PU protection (realistic)	Conflict graph	Channel allocation	Vertices—SUs pairs; edges—between SUs pairs; weights—interference	Heuristic algorithm	${\bar{R}}_{SU}$ up to 4.5 b/s/Hz, and β up to 1 for $N_{SU}=40$ and $N_{PU}=N_{c}=25$
[86]	RA in a CR IoT network/Channels of equal bandwidth (unrealistic)	Directed graph	Channel allocation	Vertices—SUs; edges—between SUs; weights—SINR	Genetic algorithm	$R_{CR,T}$ up to 20 b/s/Hz for $N_{SU}=60$
[87]	RA in an underlay CR network/Perfect CSI knowledge (unrealistic)	Interference hypergraph	Channel allocation	Vertices—SUs; edges—between SUs; weights—received power	Potential game	$\Omega_{U}=2.1$ for $N_{SU}>N_{CAP}$
[88]	RA in a CR network/PU and SU synchronization (unrealistic)	Conflict graph	Channel allocation	Vertices—SUs; edges—between SUs; weights—mean throughput	Greedy algorithm	Up to 46 dB performance loss for $N_{SU}=50$, $N_{CAP}=5$ and $N_{c}=8$

**Table 9:** Types of graphs in RA methods.

	General/Complete Graph	Bipartite Graph	Star Graph	Path Graph	GSP/GNN	References
RA methods in OFDM Systems	✗	✗				[19,20,21]
RA Methods with user and cell clustering	✗	✗		✗		[22,23,24,25,26,27]
RA methods With belief propagation	✗	✗				[28,29]
RA Methods based on GSP	✗	✗			✗	[30,31,32,33,34,43,79,80]
Joint power and channel allocation methods	✗	✗				[51,52,53]
Hypergraph-based RA methods	✗	✗				[54,55,56,57]
Graph-coloring-based RA methods	✗					[58,59,60,61]
RA methods in NOMA systems		✗				[62,63,64]
RA methods in underlay CR networks	✗	✗				[75,76,77]
RA Methods in interweave CR networks	✗	✗				[78,79,80,89]
Miscellaneous methods	✗	✗	✗	✗		[35,36,37,38,39,65,66,67,68,69,70,81,82,83,84,85,86,87,88]
Graph models appropriate for GRIST	✗	✗	✗		✗	[19,20,21,22,24,25,26,27,28,29,30,31,32,33,34,35,36,37,38,39,43,51,52,53,54,55,56,57,58,59,60,61,62,63,64,66,67,68,69,70,75,76,77,78,79,80,81,82,83,84,85,86,87,88,89,87,88]

## Data Availability

Not applicable.
